# Whole blood co-expression modules associate with metabolic traits and type 2 diabetes: an IMI-DIRECT study

**DOI:** 10.1186/s13073-020-00806-6

**Published:** 2020-12-01

**Authors:** Valborg Gudmundsdottir, Helle Krogh Pedersen, Gianluca Mazzoni, Kristine H. Allin, Anna Artati, Joline W. Beulens, Karina Banasik, Caroline Brorsson, Henna Cederberg, Elizaveta Chabanova, Federico De Masi, Petra J. Elders, Ian Forgie, Giuseppe N. Giordano, Harald Grallert, Ramneek Gupta, Mark Haid, Torben Hansen, Tue H. Hansen, Andrew T. Hattersley, Alison Heggie, Mun-Gwan Hong, Angus G. Jones, Robert Koivula, Tarja Kokkola, Markku Laakso, Peter Løngreen, Anubha Mahajan, Andrea Mari, Timothy J. McDonald, Donna McEvoy, Petra B. Musholt, Imre Pavo, Cornelia Prehn, Hartmut Ruetten, Martin Ridderstråle, Femke Rutters, Sapna Sharma, Roderick C. Slieker, Ali Syed, Juan Fernandez Tajes, Cecilia Engel Thomas, Henrik S. Thomsen, Jagadish Vangipurapu, Henrik Vestergaard, Ana Viñuela, Agata Wesolowska-Andersen, Mark Walker, Jerzy Adamski, Jochen M. Schwenk, Mark I. McCarthy, Ewan Pearson, Emmanouil Dermitzakis, Paul W. Franks, Oluf Pedersen, Søren Brunak

**Affiliations:** 1 Department of Bio and Health Informatics, Technical University of Denmark, Kongens Lyngby, Denmark; 2 Novo Nordisk Foundation Center for Basic Metabolic Research, Faculty of Health and Medical Sciences, University of Copenhagen, Copenhagen, Denmark; 3 Novo Nordisk Foundation Center for Protein Research, Faculty of Health and Medical Sciences, University of Copenhagen, Copenhagen, Denmark; 4 Center for Clinical Research and Prevention, Bispebjerg and Frederiksberg Hospital, The Capital Region, Copenhagen, Denmark; 5 Research Unit Molecular Endocrinology and Metabolism, Helmholtz Zentrum Muenchen, German Research Center for Environmental Health, Neuherberg, Germany; 6 Amsterdam UMC, location VUmc, Department of Epidemiology and Biostatistics, Amsterdam Public Health Research Institute, Amsterdam, the Netherlands; 7 Julius Center for Health Sciences and Primary Care, University Medical Center Utrecht, Utrecht, the Netherlands; 8 Department of Endocrinology, Abdominal Center, Helsinki University Hospital, Helsinki, Finland; 9 Department of Diagnostic Radiology, Copenhagen University Hospital Herlev Gentofte, Herlev, Denmark; 10 Amsterdam UMC, location VUmc, Department of General Practice, Amsterdam Public Health Research Institute, Amsterdam, the Netherlands; 11 Population Health & Genomics, School of Medicine, University of Dundee, Ninewells Hospital, Dundee, UK; 12 Genetic and Molecular Epidemiology Unit, Lund University Diabetes Centre, Department of Clinical Sciences, Clinical Research Centre, Lund University, Skåne University Hospital, Malmö, Sweden; 13 German Center for Diabetes Research (DZD e.V.), Neuherberg, Germany; 14 Research Unit of Molecular Epidemiology, Institute of Epidemiology, Helmholtz Zentrum München, Neuherberg, Germany; 15 Clinical Cooperation Group Type 2 Diabetes, Helmholtz Zentrum München and Ludwig-Maximilians Universität München, Munich, Germany; 16 Clinical Cooperation Group Nutrigenomics and Type 2 Diabetes, Helmholtz Zentrum München and Technische Universität München, Munich, Germany; 17 Novo Nordisk Research Centre Oxford, Oxford, UK; 18 Faculty of Health Sciences, University of Southern Denmark, Odense, Denmark; 19 Department of Cardiology and Endocrinology, Slagelse Hospital, Slagelse, Denmark; 20 The Institute of Clinical and Biological Sciences, University of Exeter College of Medicine and Health, University of Exeter, Exeter, UK; 21 Institute of Cellular Medicine (Diabetes), Newcastle University, Newcastle upon Tyne, UK; 22 Affinity Proteomics, Science for Life Laboratory, School of Engineering Sciences in Chemistry, Biotechnology and Health, KTH - Royal Institute of Technology, Solna, Sweden; 23 Oxford Centre for Diabetes, Endocrinology and Metabolism, University of Oxford, Churchill Hospital, Oxford, UK; 24 Institute of Clinical Medicine, Internal Medicine, University of Eastern Finland, Kuopio, Finland; 25 Wellcome Centre for Human Genetics, University of Oxford, Oxford, UK; 26 Institute of Neurosciences, National Research Council, Padova, Italy; 27 NIHR Exeter Clinical Research Facility, University of Exeter Medical School, Exeter, UK; 28 Sanofi, Diabetes Division, Research and Development, Frankfurt, Germany; 29 Eli Lilly Regional Operations GmbH, Vienna, Austria; 30 Sanofi-Aventis Deutschland GmbH, R&D, Frankfurt am Main, Germany; 31 Novo Nordisk A/S, Søborg, Denmark; 32 Department of Cell and Chemical Biology, Leiden University Medical Center, Leiden, The Netherlands; 33 Department of Epidemiology and Biostatistics, Amsterdam Public Health Institute, Amsterdam UMC, Amsterdam, The Netherlands; 34 Faculty of Health and Medical Sciences, University of Copenhagen, Copenhagen, Denmark; 35 Department of Medicine, Bornholms Hospital, Rønne, Denmark; 36 Department of Genetic Medicine and Development, University of Geneva Medical School, Geneva, Switzerland; 37 Institute of Genetics and Genomics in Geneva (iGE3), University of Geneva, Geneva, Switzerland; 38 Swiss Institute of Bioinformatics, Geneva, Switzerland; 39 Lehrstuhl für Experimentelle Genetik, Technische Universität München, Freising-Weihenstephan, Germany; 40 Department of Biochemistry, Yong Loo Lin School of Medicine, National University of Singapore, Singapore, Singapore; 41 Oxford NIHR Biomedical Research Centre, Oxford University Hospitals NHS Foundation Trust, John Radcliffe Hospital, Oxford, UK; 42 Department of Nutrition, Harvard School of Public Health, Boston, MA USA; 43 Department of Public Health & Clinical Medicine, Section for Medicine, Umeå University, Umeå, Sweden

**Keywords:** Type 2 diabetes, Transcriptomics, Co-expression modules, Omics data integration

## Abstract

**Background:**

The rising prevalence of type 2 diabetes (T2D) poses a major global challenge. It remains unresolved to what extent transcriptomic signatures of metabolic dysregulation and T2D can be observed in easily accessible tissues such as blood. Additionally, large-scale human studies are required to further our understanding of the putative inflammatory component of insulin resistance and T2D. Here we used transcriptomics data from individuals with (*n* = 789) and without (*n* = 2127) T2D from the IMI-DIRECT cohorts to describe the co-expression structure of whole blood that mainly reflects processes and cell types of the immune system, and how it relates to metabolically relevant clinical traits and T2D.

**Methods:**

Clusters of co-expressed genes were identified in the non-diabetic IMI-DIRECT cohort and evaluated with regard to stability, as well as preservation and rewiring in the cohort of individuals with T2D. We performed functional and immune cell signature enrichment analyses, and a genome-wide association study to describe the genetic regulation of the modules. Phenotypic and trans-omics associations of the transcriptomic modules were investigated across both IMI-DIRECT cohorts.

**Results:**

We identified 55 whole blood co-expression modules, some of which clustered in larger super-modules. We identified a large number of associations between these transcriptomic modules and measures of insulin action and glucose tolerance. Some of the metabolically linked modules reflect neutrophil-lymphocyte ratio in blood while others are independent of white blood cell estimates, including a module of genes encoding neutrophil granule proteins with antibacterial properties for which the strongest associations with clinical traits and T2D status were observed. Through the integration of genetic and multi-omics data, we provide a holistic view of the regulation and molecular context of whole blood transcriptomic modules. We furthermore identified an overlap between genetic signals for T2D and co-expression modules involved in type II interferon signaling.

**Conclusions:**

Our results offer a large-scale map of whole blood transcriptomic modules in the context of metabolic disease and point to novel biological candidates for future studies related to T2D.

## Background

The rising global prevalence of type 2 diabetes (T2D) is one of the major medical challenges of the twenty-first century. Despite intense research efforts, the understanding of the molecular mechanisms of this complex disease remains incomplete, hampering the development of novel therapeutic strategies and interventions. For example, it is largely unknown why some high-risk individuals remain healthy while others at apparently lower risk progress to T2D. Insulin resistance generally develops in the context of obesity and chronic low-grade inflammation [1], and while metabolism and immunity are intrinsically linked [2], the exact contribution of inflammation to the pathogenesis of insulin resistance and T2D is debated. Inflammatory markers predict incident T2D, and numerous molecular and rodent studies support that inflammation interferes with insulin signaling [3], leading to suggestions of an inflammatory contribution to the pathogenesis of T2D [4]. A recent longitudinal multi-omics study described associations between inflammatory markers and insulin resistance, and furthermore demonstrated differences between insulin-sensitive and insulin-resistant individuals in the molecular responses to stress events such as respiratory viral infections [5]. On the other hand, a Mendelian randomization study did not support a causal effect of the inflammatory marker C-reactive protein (CRP) in insulin resistance or T2D [6], although this does not exclude the possibility of a role of other upstream inflammatory effectors. Recent data suggest that some anti-inflammatory therapies may have a beneficial effect in diabetes-related traits, but further studies aimed at adding to our understanding of the inflammatory basis of diabetes and insulin resistance are called for [7].

Technological advances in the past decade have made it possible to generate high-throughput omics data in a large number of human samples, which has led to reports describing signatures of insulin resistance and T2D in serum proteomic [8, 9] and metabolomic [10–14] data that in some cases mirror insulin resistance-associated patterns in the gut microbiota [15]. By contrast, large-scale transcriptomics studies in the context of pre-diabetes and T2D are lacking, as transcriptomic studies for T2D have mainly focused on key T2D tissues such as pancreatic islets [16–19], skeletal muscle [20] and adipose tissue [21] that are not easily accessible, and those focusing on blood have been limited to small cohorts (*n* < 100) [22–28]. A recent study described whole blood transcriptomics associations with HbA1c levels in T2D patients [29], but it is still unknown if similar associations exist in people at risk but yet free of diabetes. Thus, the extent to which the transcriptional profiles of whole blood, mainly involving components of the immune system, reflect or contribute to metabolic phenotypes that predispose to T2D remains unclear.

**Fig. 1:**
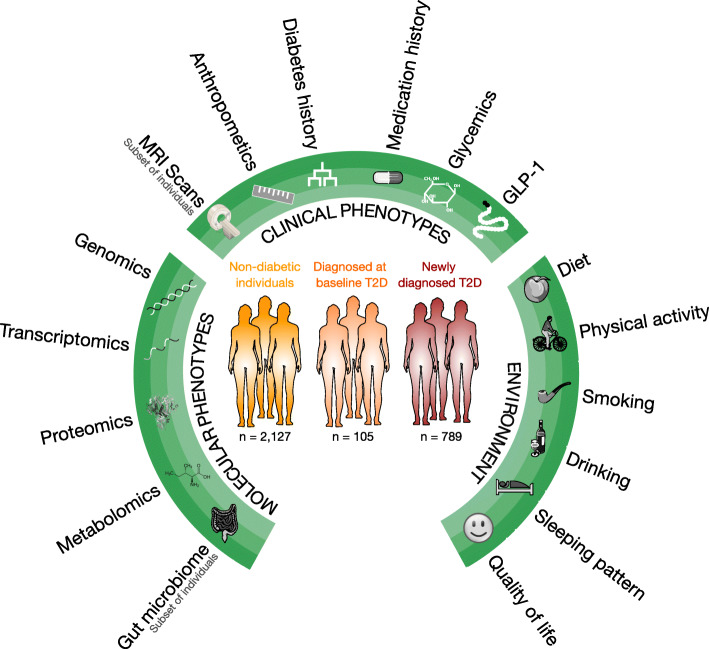
IMI-DIRECT cohort data overview. The IMI-DIRECT cohorts consist of 2127 non-diabetic individuals, 105 diagnosed-at-baseline T2D patients and 789 newly diagnosed T2D patients. All participants were deeply characterized in terms of clinical, biochemical, lifestyle and molecular phenotypes

The Innovative Medicines Intiative Diabetes Research on Patient Stratification (IMI-DIRECT) consortium generated two cohorts of deeply phenotyped non-diabetic individuals at high risk of T2D (*n* = 2127) and recently diagnosed T2D patients (*n* = 789), respectively [30, 31]. Rich multi-omics data is available in both IMI-DIRECT cohorts (Fig. 1). We here analyse this high-dimensional dataset through a data-driven dimensionality reduction approach in the context of metabolic phenotypes such as insulin resistance and glucose intolerance in IMI-DIRECT participants without diabetes (Additional file 1: Fig. S1), using a similar approach to our previous work focused on the gut microbiome [15]. By leveraging the power of clustering co-expressed genes, we identify three distinct phenotype-anchored super-modules together with a neutrophil granule protein (NGP) module that strongly associates with numerous metabolic phenotypes in non-diabetic individuals. We further explore which module associations with clinical traits are replicated in patients with T2D and which modules differ by T2D status. We describe the co-expression modules in terms of trans-omics associations and their genetic regulation and finally highlight examples of transcriptomic module rewiring by disease state.

## Methods

### Study cohorts

Data from two epidemiological cohorts that were established within the IMI-DIRECT consortium and have previously been described in detail [30, 31] were used in the current study. Briefly, the non-diabetic cohort was focused on pre-diabetes and sampled from 24,682 European-ancestry adults with available health information. Using a risk prediction algorithm, individuals at varying risk of glycaemic deterioration were identified and enrolled into a prospective cohort study undertaken at study centres in Finland (Kuopio), the Netherlands (Amsterdam), Denmark (Copenhagen) and Sweden (Lund). Of 2235 enrolled participants, 2127 passed all inclusion criteria (referred to as the non-diabetic cohort in the current study) while 105 individuals developed T2D (HbA1c ≥ 6.5%, fasting glucose ≥ 7.0 mmol/l or 2 h glucose > 11.0 mmol/l) between the enrollment and the baseline visit (referred to as the DAB-T2D group in the current study). Of the 2127 individuals, 1419 individuals presented impaired glucose control and are thus at a high risk of developing T2D. The second cohort was focused on T2D, recruiting 789 individuals with newly diagnosed T2D at study centres in the UK (Dundee, Exeter, Newcastle), the Netherlands, Sweden and Denmark. Of those, 66% received lifestyle treatment only while 34% were treated with metformin in addition to lifestyle. In the current study, we further categorized the 789 newly diagnosed T2D patients into having mild T2D (no diabetic medication, fasting glucose < 7 mmol/L and fasting HbA1c < 48 mmol/mol, *n* = 194) or more severe T2D (everyone else, *n* = 595). The clinical traits used in the current study have been described in detail elsewhere [31]. Briefly, beta cell function and glycaemic control were modeled from frequently sampled 75 g oral glucose tolerance and mixed meal tolerance tests in the two cohorts respectively. Biochemical assays were carried out centrally at the University of Eastern Finland and University of Exeter following standard protocols. Body composition was assessed using MRI and lifestyle through self-report and triaxial accelerometry. Visit date season was modeled using two trigonometric functions, sine and cosine, with a period of 1 year [32].

### Omics data

The omics data for the IMI-DIRECT cohorts used in the current study includes measurements of 119 targeted metabolites, 251 untargeted metabolites, 265 proteins (targeted by 377 antibodies) with a multiplex immunoassay and 15 proteins with a Myriad assay. Detailed information on the generation and quality control of genetic, transcriptomic, proteomic and metabolomic data for the IMI-DIRECT cohorts included in the study is provided in Additional File 2: Supplementary Methods.

### Statistical and bioinformatics analysis

#### Omics data preprocessing

Linear mixed models were fitted using transcriptomic, proteomic and metabolomic data as dependent variables and the covariates age, sex, study centre and technical variables as independent variables. The technical covariates included run date (transcriptomics, metabolomics), RNA integrity number, mean GC content and insert size (transcriptomics) and plate information (proteomics, metabolomics). The resulting residuals were rank normal transformed and used for downstream analyses.

#### White blood cell estimates

White blood cell proportions were estimated using wbccPredictor (available from https://github.com/mvaniterson/wbccPredictor). The original method used both RNAseq data and measured blood cell proportions to develop a prediction using multivariate partial-least-squares model (including age and sex). With the prediction model, the estimated cell proportions were computed and compared to the measured cell counts, which were highly correlated. The prediction model was also validated in an independent external dataset [29], which again showed high correlations with four of the five measured cell counts (neutrophils *r* = 0.81, lymphocytes *r* = 0.83, monocytes *r* = 0.64, eosinophils *r* = 0.91 and basophils *r* = 0.08).

#### Definition of co-expression modules

The preprocessed gene expression data was initially checked for potentially outlying individuals. Specifically, we declared individuals as outliers if they were > 2.5 standard deviations away from the mean connectivity to other samples in a signed biweight midcorrelations sample-sample network. No individuals were classified as outliers using these criteria. Clusters of co-expressed genes were identified in the non-diabetic cohort using the R package WGCNA [33]. Signed, weighted gene co-expression correlation (biweight midcorrelations, with a maximum 5% of the individuals regarded as outliers (maxPOutliers = 0.05)) networks were calculated across included individuals using all pairwise observations. A scale-free topology criterion (*R*^2^-cutoff for scale-free topology = 0.8) was used to choose the soft threshold (resulting in *β* = 15). Clusters of positively correlated genes were identified with the dynamic hybrid tree-cutting algorithm, using a deepSplit of 4, a minimum cluster size of 10 and the partitioning around medoids option turned on. The expression patterns of each gene module were summarized by the module eigenvector (that is, the first principal component of the gene expression across individuals). Pairs of modules were subsequently merged if the correlation between the modules’ eigenvectors exceeded 0.85. Genes that did not fit the clustering criteria were combined in a leftover group named ‘M0’. Enrichment analysis of tissue-specific gene expression of genes in modules M1-M55 compared to genes in the M0 cluster was performed using TissueEnrich [34], using the full set of 16,209 genes passing QC in the IMI-DIRECT transcriptomic data as background.

#### Module stability

To assess whether the resulting co-expression modules were robustly defined in the non-diabetic cohort, we performed a subsampling analysis, where the network construction and module identification were repeated 100 times (Additional file 1: Fig. S2) using the same parameters while only including randomly drawn 63% of the individuals, as implemented in the ‘*sampledBlockwiseModules*’ function in the WGCNA R package [33]. For each gene, the consistency was calculated as the percentage of the 100 subsamplings where the gene was assigned to the same module as when using all 2127 non-diabetic individuals. Finally, the stability of each module was defined as the average gene consistency of all genes constituting the given module.

Among the co-expression modules, we observed a number of ‘super-modules’, i.e. clusters of internally correlated modules. When calculating super-module consistency, we considered all genes assigned to any of the modules within a given super-module (defined using all 2127 non-diabetic individuals). For each of these genes, we first calculated the super-module consistency as the percentage of ‘eligible’ subsamplings where the gene was assigned to one of the modules constituting the given super-module. Some of the 100 subsamplings resulted in new modules not observed in the full cohort, e.g. if a module was split into two. As we do not know whether such new modules would fall within a given super-module, we defined eligible subsamplings as either those where the gene was assigned to (i) one of the 55 modules or unclustered or (ii) one of the 55 modules (i.e. also excluding subsamplings where the gene was unclustered). Finally, the super-module stability of each of the three super-modules was defined as the average gene super-module consistency of all genes constituting the given module.

#### Immune cell enrichment

Independent enrichment of gene modules for transcriptomic signatures of immune cell types was performed using the Human Immune Cell Transcriptome dataset (GSE3982) [35] obtained from the NCBI Gene Expression Omnibus (GEO). Using the online NCBI GEO2R tool, we performed differential expression analysis comparing each cell type to all other immune cell types within the data set (basophils, mast cells, eosinophils, dendritic cells, macrophages, neutrophils, B cells, effector memory T cells, NK cells, central memory T cells, Th1 cells, Th2 cells). The log2-fold change-ranked gene lists formed the comparative signatures of the immune cell types. We tested the enrichment of module genes within these ranked gene lists using the ‘gage’ generally applicable gene-set enrichment Bioconductor package.

#### Functional annotation of co-expressed modules

We downloaded 4319 biological pathways from ConsensusPathDB release 32 [36]. Over-representation analysis of pathways was tested using a hypergeometric test. In short, all pathways with at least two genes from the given module were tested. The background was restricted to the subset of all 16,209 genes within the gene expression data that participate in at least one pathway (in total 9270 genes), and similarly, only module genes that were part of the background were included for testing.

#### Phenotypic and trans-omics characterization of co-expressed modules

To remove potentially confounding effects in a preprocessing analysis, a linear model including age, sex and study centre as independent variables was fitted for all clinical traits (dependent variable), and the resulting residuals rank normal transformed and used for analysis. Associations between modules and continuous clinical traits, metabolites and protein residuals were assessed by linear regression models, using the module eigenvectors as independent variables. In secondary analyses, estimated white blood cell counts and visit date season were included as covariates in the models. Associations with a Benjamini-Hochberg false discovery rate (FDR) < 0.05 were considered significant, while collectively considering the full set of modules and phenotypes tested. Associations with T2D status were tested with logistic regression on the rank normal transformed and residualized data and adjusted for multiple hypothesis testing with a Benjamini-Hochberg FDR.

#### Module preservation

To further validate the co-expression modules, we assessed whether modules identified in the non-diabetic cohort were also preserved in the newly diagnosed T2D cohort. However, modules identified in non-diabetic individuals but not preserved in T2D patients are also potentially interesting as they may represent rewired pathways dysregulated or disrupted in the disease state. Specifically, we applied the module preservation analysis from the WGCNA framework where, in short, multiple different preservation statistics (evaluating both module density and intramodular connectivity) were calculated and their significance evaluated using gene-permutations resulting in *Z*-scores (200 permutations). These *Z*-scores were finally aggregated into a single combined preservation measure for each co-expression module (*Z*_summary_), where *Z*_summary_ *= (Z*_density_ *+ Z*_connectivity_)/2. General guidelines (originating from simulation studies) state: *Z*_summary_ > 10, strong evidence for preservation; 2 < *Z*_summary_ < 10, weak to moderate evidence for preservation; *Z*_summary_ < 2, no evidence for preserved. However, as *Z*_summary_ tends to increase with module size the composite *medianRank* statistic (here the multiple different preservation statistics are aggregated in a single score using the median of their individual ranks) is also provided for comparing the relative preservation between modules of different size, where lower *medianRank* indicates stronger preservation. For further details, we refer to [37]. In the preservation analysis, modules with > 1000 genes were reduced by randomly sampling 1000 genes.

#### Module-QTL analysis

Module-QTL analysis was performed to identify loci associated with the expression profiles of the 55 modules. The analyses were performed with SNPTEST version 2.5 [38] based on an additive model and by using the frequentist approach implemented in the tool. The missing data likelihood score test was used to control for genotype uncertainty. For each of the 55 modules, SNPTEST was used to evaluate the association between module and genotyped/imputed SNPs with MAF > 0.05 (*n* SNPs = 6,066,827) from the combined sample of non-diabetic and newly diagnosed T2D individuals (*n* = 2914). A study-wide significance threshold was defined as *P* < 0.05/ 6,066,827 SNPs = 8.2 × 10^− 09^. The first three principal components of the genotype data were regressed out from each module to account for population stratification and the inverse normal transformations of the residuals were used in the analysis. Module-QTLs were selected after Bonferroni correction controlling for a family wise error rate at 0.01. LocusZoom version 1.4 [39] was used to visualize the module-QTLs by plotting a region of 1 MB flanking the reference SNPs (SNP with lowest *P* value). If there were more than one reference SNPs (same *P* value) in the same genomic region, the window was computed around the first and the last SNP and the central SNP was labeled as reference. The genome-wide recombination rates were estimated from phased haplotypes in HapMap Release 22 (NCBI 36), and previous GWAS information were retrieved from the GWAS catalog and filtered to SNPs with a *P* value < 5 × 10^− 08^. Linkage disequilibrium was computed using the genotype data of the combined sample of non-diabetic and newly diagnosed T2D individuals in IMI-DIRECT. The module-QTLs were compared with the results from the DIAMANTE T2D GWAS [40]. LocusZoom plots were generated by superimposing summary statistics of the module-QTLs and of the full DIAMANTE T2D GWAS meta-analysis (MAF > 0.05) with and without adjustment for BMI. Colocalization analysis was performed using the ‘coloc’ R package [41] with default priors.

#### Module rewiring

Rewiring of co-expression modules (defined from non-diabetic individuals) in patients with diabetes was here assessed using the change in intra-module correlation structure between the discovery and newly diagnosed T2D cohorts, which further allows straightforward visualization. Specifically, we summarized a module’s correlation structure by the average biweight midcorrelation between any two genes constituting the module (i.e. ignoring the diagonal/self-interactions). Networks depicting the correlation structure in the discovery and newly diagnosed T2D cohorts and the difference between the two were made using igraph [42]. In addition to networks based on correlation coefficients calculated using all individuals in the given cohort (*n* = 2127 and *n* = 789), we also generated networks using an equal subset of individuals (*n* = 2/3 × 789 = 526) to circumvent any potential biases originating from cohort size differences. For the latter, the edge strengths are the average biweight midcorrelation coefficient from 50 subsamplings.

## Results

The study workflow is overviewed in Additional file 1: Fig. S1. In our discovery analysis, we used data from 2127 non-diabetic northern European IMI-DIRECT participants at high risk of developing T2D [31]. In a secondary analysis, two distinct IMI-DIRECT T2D patient groups were included; (a) 105 participants who were recruited to the non-diabetic cohort based on a screening visit but had a diabetic oral glucose tolerance test (OGTT) result at the baseline visit, hereafter referred to as the diagnosed-at-baseline (DAB)-T2D cohort and (b) 789 patients recruited directly into the IMI-DIRECT T2D patient cohort, hereafter referred to as the newly diagnosed (ND) T2D cohort. The baseline characteristics of the cohorts are shown in Additional file 3: Table S1 and have been described in detail elsewhere [31].

### Definition and characteristics of whole blood co-expression modules

We used whole blood RNAseq data within which we identified modules of co-expressed genes. We applied a weighted gene correlation network analysis (WGCNA) [33] to define functional transcriptomic co-expression modules. After adjusting for age, sex, study centre, RNA integrity number, mean GC content and insert size, we identified 55 modules of co-expressed genes in the 2127 individuals without diabetes (ranging in size from 11 to 1248 genes), in addition to the 6456 unclustered genes in module M0 (Additional file 1: Fig. S3, Additional file 3: Table S2). To better understand the overall co-expression module structure, we performed an enrichment analysis of tissue-specific gene expression using data from GTEx [43], Human Protein Atlas [44] and ENCODE [45], which revealed a clear distinction for clustered versus unclustered genes. The clustered genes were only enriched for genes with tissue-specific expression in immune tissues such as spleen, lymph node, appendix and bone marrow, while the unclustered genes were enriched for tissue-specific genes from almost all other tissues and depleted for genes with enhanced expression in immune tissues (Additional file 1: Fig. S4). Thus, the transcriptomic modules observed in whole blood are mainly driven by the immune system, whereas genes of major importance in other tissues are generally absent from this structure, even though expressed in blood. Pathway enrichment analysis of the co-expression modules revealed functional diversity (Additional file 3: Table S3), where examples of strongly enriched pathways within modules included ‘Type II interferon signaling’ (M29, *q* = 1.8 × 10^− 12^), ‘Neutrophil degranulation’ (M8, *q* = 5.0 × 10^− 10^; M35, *q* = 1.3 × 10^− 24^), ‘Erythrocytes take up oxygen and release carbon dioxide’ (M30, q = 1.5 × 10^− 08^), ‘Platelet activation, signaling and aggregation’ (M18, q = 8.0 × 10^− 19^), and ‘B-Cell antigen Receptor (BCR) pathway’ (M17, *q* = 8.3 × 10^− 10^). Thus, the whole blood transcriptomic modules capture functionally related genes that in some cases represent cell-type-specific processes.

**Fig. 2:**
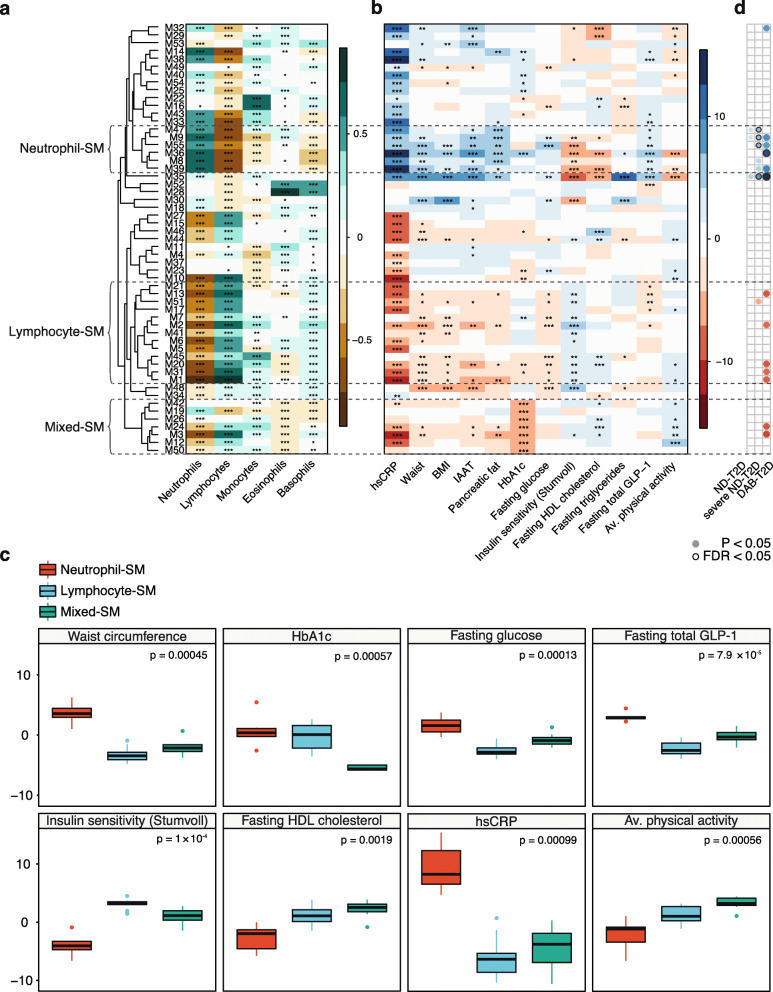
Whole blood transcriptomic co-expression modules show extensive correlations with clinical traits in 2127 non-diabetic individuals. **a** Heatmap showing Pearson’s correlation between modules (rows) and white blood cell estimates. Stars indicate statistical significance as such: ***FDR < 0.001, **FDR < 0.01, *FDR < 0.05. **b** Heatmap providing overview of the associations between modules and selected clinical traits. The heatmap colors denote the linear regression estimates, where all phenotypes have previously been rank normal transformed and residualized for age, sex, centre and technical covariates. **c** Boxplots demonstrating the distinct clinical trait associations for the three transcriptomic super-modules, as the average estimate for associations across all modules within a given super-module. *P* values are shown for Kruskal-Wallis rank sum test between the three super-modules. **d** T2D associations for each module where the colour indicates a positive (blue) or negative association (red). SM, super-module; IAAT, intra-abdominal adipose tissue

Among the co-expression modules, we observed a number of ‘super-modules’ (SMs) (Additional file 1: Fig. S5), defined as clusters of internally correlated modules, which as a whole were highly stable (super-module stability estimate > 79%, Additional file 3: Table S4). As white blood cell (WBC) counts were not available in our data, we estimated WBC proportions per individual from the transcriptomics data as previously described [29] and investigated whether the relative expression of the genes forming the co-expression modules was reflective of differences in WBC proportions. We observed strong and consistent correlations between two super-modules and estimated neutrophil and lymphocyte levels, respectively (Fig. 2a), hereafter referred to as the neutrophil-SM and lymphocyte-SM, which were inversely correlated (Additional file 1: Fig. S5). Separate immune cell signature enrichment analysis of the modules using the Human Immune Cell Transcriptome data [35] corroborated these findings by revealing an enrichment of neutrophil gene signatures among modules in the neutrophil-SM, and while we observed more mixed cell-type enrichment for modules in the lymphocyte-SM modules, these mainly included lymphocyte gene signatures (Additional file 3: Table S5). The six modules within the neutrophil-SM were consistently enriched for pathways involved in inflammatory mechanisms and response to infections (Additional file 3: Table S3). Thus, these two super-modules seem to a large extent to reflect the neutrophil/lymphocyte ratio in whole blood. Other modules with clear cell-type-specific signatures included module M3, which was enriched for T cell gene expression signatures, and modules M16 and M22, which were enriched for monocyte signatures (Additional file 3: Table S5). Yet, these two monocyte modules were notably different in a pathway enrichment analysis that revealed an enrichment of lysosomal genes in M16 (*q* = 5.7 × 10^− 13^) and pathways such as sphingolipid metabolism (*q* = 0.034) and vesicle-mediated transport (*q* = 0.034) for M22 (Additional file 3: Table S3). While these examples demonstrate cell-type-specific clustering of transcripts, we also observed that many modules were enriched for multiple cell-type signatures, indicating that they are not solely driven by the abundance of specific cell types (Additional file 3: Table S5).

### Whole blood co-expression module associations with clinical traits in individuals without diabetes

To investigate the overall relationship between whole blood transcriptomic profiles and metabolic clinical traits in 2127 individuals without diabetes, we characterized the 55 co-expression modules in terms of their associations with clinical phenotypes. We observed numerous associations between transcriptomic modules and measurements related to inflammation, fat tissue, glucose tolerance, insulin sensitivity, lipids and physical activity (Fig. 2b, Additional file 1: Fig. S6, Additional file 3: Tables S6-S7), thus revealing a major overlap between metabolic and immune processes. Not surprisingly, most modules (42/55 = 76%) were significantly associated with CRP levels, indicating that most whole blood transcriptomic modules are affected by inflammatory status. By contrast, fewer signals were detected overall for measures of beta cell function, incretin levels or diet (Additional file 1: Fig. S6, Additional file 3: Tables S6-S7), suggesting that these traits are not strongly reflected in whole blood transcriptomic modules, given the clinical and diet measurements used in this study.

Three super-modules showed distinct clinical trait association profiles. The neutrophil-SM was characterized by consistent negative associations with insulin sensitivity and positive associations with CRP levels, abdominal and pancreatic fat and fasting GLP-1 levels (Fig. 2c). The lymphocyte-SM generally showed clinical associations that contrasted those that characterized the neutrophil-SM (Fig. 2b, c, Additional file 3: Tables S6-S7). Finally, a third super-module with a mixed cell-type association profile (Fig. 2a), hereafter referred to as the mixed-SM, exhibited a strong negative association with fasting HbA1c and positive association with physical activity and examination date, indicating seasonal variation of the seven composite modules (Fig. 2b, c, Additional file 1: Fig. S6). These seven modules were enriched for diverse functions, such as chromatin organization, gene expression and signaling pathways, including EGF-EGFR, PI3K/AKT and TGF (Additional file 3: Table S3).

**Fig. 3:**
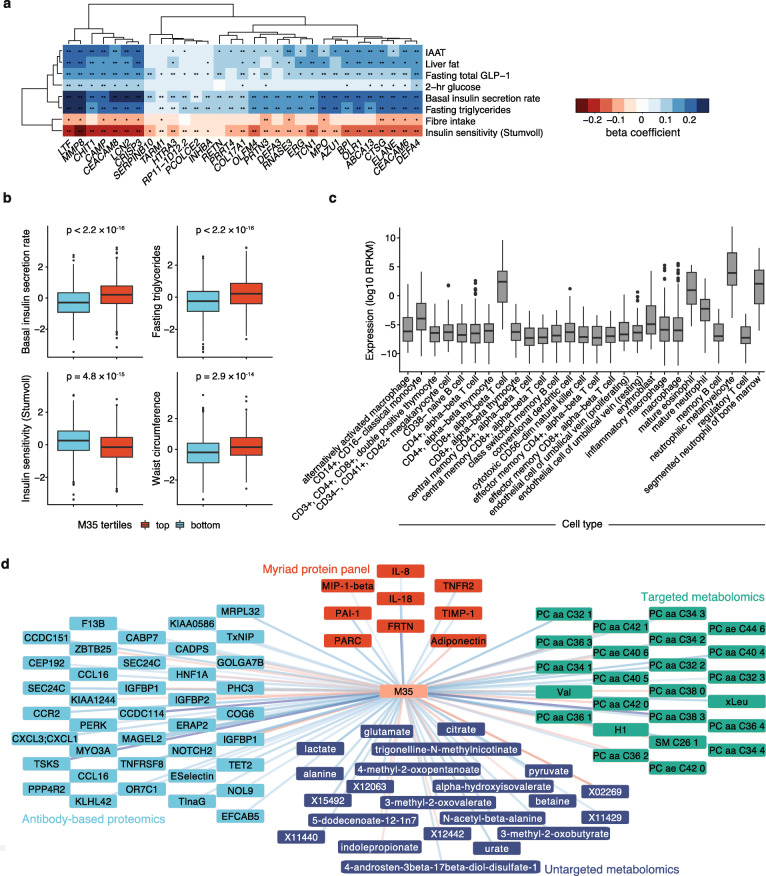
NGP module M35 clinical and cross-omics associations and expression profile across immune cell types. **a** Heatmap demonstrating the associations between individual genes in module M35 and selected clinical traits. The heatmap colors denote the linear regression estimates, where all phenotypes have previously been rank normal transformed. Stars indicate statistical significance as such: ***FDR < 0.001, **FDR < 0.01, *FDR < 0.05. **b** Individuals within the top (red, *n* = 709) and bottom (blue, *n* = 709) tertiles of M35 expression have significantly different insulin secretion rate, triglycerides, insulin sensitivity and waist circumference. *P* values are shown for a two-sided *t*-test comparing rank normal transformed variables between the two groups. **c** Average expression of M35 genes across haematopoietic cell types from the BLUEPRINT consortium. **d** Cross-omics associations for module M35, including features from untargeted metabolomics (dark blue), targeted metabolomics (green), antibody-based proteomics (light blue) and the Myriad protein panel (red). The edge colour indicates a positive (blue) or negative (red) direction of effect for the given association. IAAT, intra-abdominal adipose tissue

Apart from the three highlighted super-modules, one independent module (M35) stood out due to its strong associations with multiple clinical traits compared to all other modules (Fig. 2b and Fig. 3a, b, Additional file 1: Fig. S6, Additional file 3: Tables S6-S7). These included positive associations with basal insulin secretion rate (FDR = 3.76 × 10^− 26^), total GLP-1 (FDR = 2.19 × 10^− 09^), 2-h glucose (FDR = 2.56 × 10^− 04^), intra-abdominal adipose tissue (FDR = 9.28 × 10^− 08^) and liver fat (FDR = 3.14 × 10^− 06^), and negative associations with insulin sensitivity (Matsuda, FDR = 3.93 × 10^− 19^; 2 h OGIS, FDR = 1.4 × 10^− 13^) and fiber intake (FDR = 4.17 × 10^− 04^). We performed additional multivariate analyses for module M35 and selected clinical traits and found the associations between M35 and insulin sensitivity (Matsuda), BMI and GLP-1 to be largely attenuated after adjustment for basal insulin secretion rate, whereas the associations of M35 with basal insulin secretion rate, fasting triglycerides and CRP remained strongly significant in all models tested (Table 1, Additional file 3: Table S8). Module M35 was extremely stable within the non-diabetic cohort (stability estimate = 98%, indicating that on average each gene was retained within the module in 98% of random subsamples, Additional file 3: Table S4 and “Methods”) and did not correlate strongly with any other module (maximum Pearson’s *r* = 0.23 for M33, Additional file 1: Fig. S5). M35 contained 31 genes, most of which encode neutrophil granule proteins (NGPs) with antibacterial properties that play a role in the innate immune system, and overlapped extensively with a previously described neutrophil co-expression module in blood [46] (Additional file 3: Table S3 & S9). In BLUEPRINT gene expression data from various blood cells [47], we found the M35 genes to be highest expressed in neutrophil precursors (Fig. 3c), but intriguingly, further immune cell signature enrichment analysis using the Human Immune Cell Transcriptome data [35] revealed that many M35 genes (*CEACAM8*, *LTF*, *ELANE*, *MPO*, *BPI* and *CTSG*) are in fact more highly expressed in mast cells compared to neutrophils (Additional file 3: Tables S5 & S10). As expected, the individual genes in M35 showed consistent directions of effects for their clinical associations, with the strongest associations observed for *CRISP3*, *CAMP*, *LTF*, *MMP8*, *LCN2* and *CEACAM8* (Fig. 3a). Here, the latter four were the most central genes within the M35 module together with *CEACAM6* and *DEFA4*, as indicated by the intramodular connectivity parameter (kIM, Additional file 3: Table S9), and can as such be considered as hubs within the module. After additional adjustment for WBC proportions and examination date, many associations remained highly significant, such as for the NGP module M35 and the mixed-SM (Additional file 1: Fig. S7).

**Table 1:** Associations between module M35 and six selected clinical traits in multivariate regression models where adjusting for the other five variables

	Basic model (DV ~ M35 + age + sex + study centre + RNAseq technical variables)			Basic model + other 5 variables		
Dependent variable (DV)	Beta	s.e.	*P*	Beta	s.e.	*P*
Basal insulin secretion rate	10.75	0.95	6.83 × 10^−29^	1.92	0.50	1.23 × 10^−04^
Matsuda insulin sensitivity index	−9.20	0.96	1.67 × 10^−21^	0.55	0.51	0.28
Triglycerides	10.06	0.96	4.67 × 10^−25^	5.01	0.89	2.17 × 10^−08^
Body mass index	7.24	0.98	1.60 × 10^−13^	0.40	0.85	0.63
hsCRP	7.68	0.96	2.42 × 10^−15^	5.06	0.96	1.53 × 10^−07^
Total GLP-1	6.40	0.96	2.99 × 10^−11^	1.94	0.91	0.03

*DV* dependent variable, *GLP-1* glucagon-like protein 1, *hsCRP* high-sensitivity C-reactive protein, *s.e.* standard error

In summary, we identified extensive associations between whole blood transcriptomic profiles and metabolic traits in non-diabetic individuals, the strongest of which were observed between the NGP module M35 and measures of abdominal obesity and insulin resistance. In addition, we identified three major transcriptomic super-modules, two of which exhibit inflammatory and WBC-proportion-mediated associations with central obesity and leanness, respectively, while the third was strongly associated with lower fasting HbA1c levels, independently of both WBC proportions and seasonal variation in whole blood gene expression.

### Co-expression module associations in T2D patients

To validate the importance of the identified transcriptomics signatures of insulin resistance and glucose intolerance in individuals without diabetes, we investigated the associations between the transcriptomic modules and clinical traits in the newly diagnosed T2D cohort (*n* = 789). We found all 55 transcriptomics modules to be significantly preserved (see “Methods”) between the non-diabetic and the newly diagnosed T2D cohort (Additional file 1: Fig. S8), thus validating them as biologically relevant entities that can be compared between groups. Overall, we observed fewer associations between the modules and clinical traits in the 789 T2D patients compared to the 2127 non-diabetic individuals (Additional file 1: Fig. S9), possibly because of the smaller cohort size and differences in phenotype variance, and therefore reduced power to detect the associations. However, the associations between the NGP module M35, which was highly preserved in the newly diagnosed T2D cohort (medianRank = 5, Additional file 3: Table S4 and “Methods”), and adiposity, insulin sensitivity, fasting glucose and triglycerides were replicated in the newly diagnosed T2D cohort. Similarly, for the neutrophil- and lymphocyte-SMs, numerous associations with insulin sensitivity, glucose tolerance and adiposity were validated in the newly diagnosed T2D cohort. Conversely, while directionally consistent, the association between the mixed-SM and HbA1c levels in T2D patients was not significant (Additional file 1: Fig. S9). We further investigated if the lack of statistical significance was due to confounding of medication use in the newly diagnosed T2D cohort but obtained similar results after adjusting or stratifying by metformin use, observing only a slight increase in statistical significance in T2D patients on metformin (Additional file 3: Table S11).

We next compared the relative expression of the co-expression modules in the non-diabetic individuals to both the newly diagnosed T2D and DAB-T2D patient groups, as well as to only the more severely diabetic newly diagnosed T2D patients (see “Methods”). We found five modules to differ (Student’s *t* test, FDR < 0.05) between non-diabetic and T2D individuals in one or more of the three comparisons; four members (M9, M36, M47, M55) of the neutrophil-SM and the NGP module M35, which differed most between non-diabetic individuals and T2D patients of all 55 modules (FDR_DAB-T2D_ = 0.016, FDR_ND-T2D_severe_ = 4.0 × 10^− 03^) (Additional file 3: Table S12, Fig. 2d). The directions of effect for module associations with T2D were consistent with the clinical trait associations previously observed within the non-diabetic cohort, as they were all higher expressed in T2D patients than non-diabetic individuals (Additional file 3: Table S12).

### Trans-omics associations

To shed light on potential molecular mechanisms involved in the transcriptomic module associations with clinical traits in non-diabetic individuals, we utilized the rich multi-omics data in IMI-DIRECT to further describe the transcriptomic modules in terms of their associations with metabolomic and proteomic measurements. For this analysis, we included measurements of 119 targeted metabolites, 251 untargeted metabolites, 265 proteins (targeted by 377 antibodies) with a multiplex immunoassay and 15 proteins with a Myriad assay. We observed 7521 significant (FDR < 0.05) trans-omics associations for the transcriptomic modules in the non-diabetic cohort, and as expected due to differences in sample size, fewer in the ND-T2D cohort, or 1414 associations (Additional file 1: Fig. S10a). Of the 7521 trans-omics associations detected in the non-diabetic cohort, 2459 (33%) were replicated in the newly diagnosed T2D cohort (*P* < 0.05) where 2412 (98%) were directionally consistent between the two (Additional file 1: Fig. S10b-c, Additional file 3: Table S13).

We noted that the NPG module M35, of particular interest due to its strong clinical associations, was the module with the highest number of unique omics associations, i.e. it was associated with 25 omics measurements that were not associated with any other module. These included a negative association with the gut microbiota-produced metabolite indolepropionate and positive associations with the BCAAs valine and leucine (Fig. 3d, Additional file 3: Table S13). Module M35 was furthermore associated with BCAA breakdown products, such as 3-methyl-2-oxobutyrate, and numerous phosphatidylcholines (Fig. 3d, Additional file 3: Table S13).

### Genetic regulation of whole blood transcriptomic modules

**Fig. 4:**
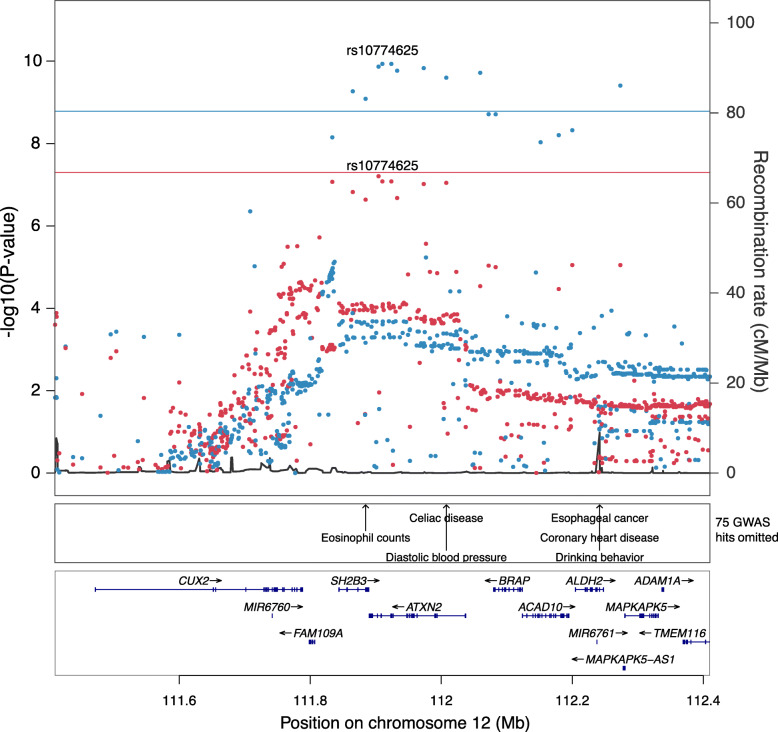
LocusZoom plots for module M23 and T2D associations on chromosome 12. Associations for module M32 in IMI-DIRECT (blue) are shown together with T2D associations (red) in the same region from the DIAMANTE GWAS. The lines denote a genome-wide significance threshold of *P* = 5 × 10^− 08^ (red line) and the IMI-DIRECT study-wide significant threshold of *P* = 8.2 × 10^− 09^ (blue line)

To better understand the regulatory mechanisms underlying the structure of the co-expression modules in whole blood, we performed a genome-wide association analysis for each module to identify genetic variants associated with module expression, or module quantitative trait loci (module-QTL). We performed the module-QTL analysis in both the non-diabetic cohort alone (*n* = 2127) and in the combined sample of participants with and without T2D (*n* = 2914). Results from the two different module-QTL analyses were highly concordant (Additional file 1: Fig. S11), and we therefore continued with the combined sample for increased statistical power. For nine modules, we identified one or more study-wide significant (*P* < 8.2 × 10^− 09^) module-QTLs (Additional file 3: Table S14), where one locus on chromosome 12 was associated with two modules (M29 and M32) consistent with these modules being correlated (Pearson’s *r* = 0.78). The modules with a module-QTL tended to be small (range 13–155 genes, mean = 44 genes) and highly stable (mean stability estimate = 87%, SD = 16%, Additional file 3: Table S4 and “Methods”). As the majority of the co-expression modules were associated with glycemic traits such as insulin resistance, glucose intolerance and T2D in our observational analysis, we performed a lookup of the 17 module-QTL lead variants in summary statistics from the DIAMANTE T2D genome-wide association study (GWAS) in 898,130 European-descent individuals [40]. At a Bonferroni-corrected *P* value threshold (*P* < 0.05/17 module-QTLs = 0.0029), we found the genetic signal for modules M32 and M29 on chromosome 12 to be associated with T2D and T2D adjusted for BMI (Additional file 3: Table S14). A colocalization analysis supported a shared causal variant underlying these traits (posterior probability = 99.1%, Fig. 4). The lead variant for modules M32 and M29 in this locus are in strong LD (*r*^2^ > 0.94) with the nonsynonymous *SH2B3* variant rs3184504, which is highly pleiotropic and has been associated with numerous immune, haematologic and metabolic traits [48–50]. A lookup of the M32 lead variant rs10774625 in publicly available GWAS summary statistics revealed similar links, including genome-wide significant associations with cholesterol, coronary artery disease, HbA1c, serum urate and hypothyroidism (Additional file 1: Fig. S12). Both modules M32 and M29, regulated by this locus in our study, were strongly enriched for type II interferon signaling, NOD-like receptor signaling and other immune pathways (Additional file 3: Table S3). Of note, the three module-QTLs identified for module M35 were not significantly associated with T2D, thus suggesting a non-causal relationship between this module and T2D.

### Module rewiring in T2D

The transition between healthy and complex disease states may follow, or lead to, the rewiring of molecular networks, driving phenotypic changes [51]. Weak network or module preservation between disease cases and controls may indicate context-specific regulation, and we hypothesized that an altered co-ordination or rewiring of a functional module might take place in a disease state such as T2D. While we found all co-expression modules to be preserved to some extent between the non-diabetic and T2D individuals (Additional file 1: Fig. S8), they could still be ranked in terms of preservation. We calculated module rewiring between the non-diabetic and newly diagnosed T2D cohort, defined as the change in absolute mean correlation between genes within a given module (“Methods”). Here, the most noteworthy module was module M30, which was almost perfectly stable within the non-diabetic cohort (stability estimate = 97%) while being one of the modules least preserved between cohorts (medianRank = 52) and having lower connectivity within the newly diagnosed T2D patients (Additional file 3: Table S4), suggesting its rewiring is very specific to the diabetic state. This module was strongly enriched for erythrocyte pathways such as ‘Erythrocytes take up oxygen and release carbon dioxide’ (FDR = 1.5 × 10^− 08^) and was positively associated with adiposity, insulin resistance and fasting triglycerides in non-diabetic individuals (Fig. 2a). The module showing the largest extent of rewiring from non-diabetic to T2D individuals was M26 (Additional file 1: Fig. S13, Additional file 3: Table S4), which was also the least preserved module (medianRank = 54, Additional file 3: Table S4 and “Methods”). This module, which was enriched for Rho GTPase and TGF signaling (Additional file 3: Table S3), had considerably fewer intra-module correlations in the newly diagnosed T2D patients compared to the non-diabetic individuals (Additional file 3: Table S4). Module M26 was a member of the mixed-SM, showing a negative association with fasting HbA1c levels and a positive association with fasting HDL levels and physical activity in the non-diabetic cohort (Fig. 2).

## Discussion

The current study is the largest study to date to describe transcriptomic signatures of metabolic clinical traits in non-diabetic individuals and T2D patients and demonstrates that whole blood transcriptomic modules, which mainly describe co-expression within immune cells, strongly reflect metabolic health. We identify transcriptomic signatures of insulin resistance and glucose intolerance in non-diabetic individuals, many of which we replicate in T2D patients. These signatures are partly driven by neutrophil/lymphocyte ratios while others are independent of WBC proportions, including those of the NGP module M35. Furthermore, five transcriptional modules associated with quantitative metabolic traits in non-diabetic individuals were differentially expressed in T2D patients compared to those without diabetes. From a clinical perspective, our findings highlight the potential for further investigation of certain immune cell subpopulations in relation to metabolic health, which could provide more economically feasible biomarker options for clinical practice than whole blood transcriptomics.

Previous studies have demonstrated extensive network structure between genetics, transcripts and metabolites in whole blood that have been described as an interface between inflammation and metabolism [46, 52]. Here we apply similar approaches to investigate the relevance of such network structures to human clinical traits related to metabolism and T2D. Co-expression analysis differs from the analysis of physical interactions in the sense that transcriptional correlation can be caused by numerous different mechanisms, including shared regulation at the transcriptional or post-transcriptional level, but also factors such as proportion of cell types in a given tissue. A drawback of module analysis is that some fine-grained information is lost, and genes that are not part of any module are excluded from the analysis, yet this approach also reduces noise in large-scale data and facilitates the discovery of shared patterns across functionally related genes.

The strongest signals observed in our study were for the NPG module M35, which was associated with a wide range of clinical traits in non-diabetic individuals, even independently of estimated WBC proportions. A module overlapping with module M35 has been previously described and linked to serum metabolomics profiles [46], and we here extend these findings by demonstrating the strong clinical relevance of this module. We furthermore find that module M35 is the only transcriptomic module in blood that correlates with serum levels of BCAAs in our data and the gut microbiota-produced metabolite indolepropionate. BCAAs are established metabolite markers for risk of T2D [14], and we have previously shown in another cohort that the BCAAs co-occur with metabolites related to gut microbial metabolism [15]. These associations raise the question if the module M35 might be related to gut microbial composition. Module M35 mainly consists of genes encoding granule proteins with antibacterial properties, and while many of these have established roles in neutrophils, our results suggest they may be important in mast cells as well. The transcriptional regulation of NGPs takes place during the cell differentiation process in the bone marrow [53], consistent with our observation that these genes are highest expressed in neutrophil precursors. This might indicate that the transcriptional levels of these genes observed in peripheral blood represent a certain cell subpopulation, possibly immature neutrophils, rather than a transcriptional response. The presence of immature neutrophils in blood (generally termed ‘left shift’) occurs for example as a response to inflammation due to bacterial infections [54]. Infection burden and metabolic endotoxemia are linked to insulin resistance [55, 56], and further studies will be required to elucidate if the NGP module plays a role in the immune response to such conditions. To our knowledge, this gene module is novel in the context of insulin resistance and T2D and while our data do not support an independent causal role of it in T2D aetiology, it is an interesting candidate for further mechanistic studies.

Overall, we find that the co-expression structure of whole blood transcriptomic data to a large extent reflects WBC proportions. This observation is in line with previous reports on much of the variance of blood transcriptomics being explained by inter-individual differences in the proportions of WBC subtypes [57]. A limitation of our study is the lack of measured blood cell counts, thus restricting us to the use of estimated white blood cell proportions from the transcriptomics data. However, in a previous study, the same types of estimates were highly correlated with measured cell proportions in external data independent of the training data [29]. Furthermore, in the current study, additional pathway and immune cell-type enrichment analyses corroborated the conclusions derived from using these estimates. The impact of WBC proportions on whole blood transcriptomics data in our study is mainly reflected in the two super-modules that to a large extent seem to represent neutrophil and lymphocyte proportions and associate with central obesity and leanness, respectively. Neutrophil/lymphocyte ratio is a general marker of low-grade inflammation and has been suggested as a biomarker for cardiac mortality [58] and cancer prognosis [59]. The extensive associations between these super-modules and clinical traits in our results suggest that the utility of the neutrophil/lymphocyte ratio should be studied further in the context of T2D, especially as total WBC counts are predictive of incident T2D [60, 61]. The seasonal variation of the third super-module, the mixed-SM, suggests its composite genes may be involved in the regulation or response to biological rhythms or environmental exposures. Yet, this super-module remained associated with HbA1c levels after adjustment for both visit date and estimated WBC proportions, suggesting these genes may also be directly related to glucose homeostasis. However, these associations were not replicated in the newly diagnosed T2D cohort, a finding that was not explained by confounding from anti-diabetic medication use and indicates that the observed associations between the mixed-SM and HbA1c may not be directly transferable to T2D patients.

Integrating genetic data, we found the highly pleiotropic *SH2B3* locus, which overlaps with a nominal T2D signal from the DIAMANTE GWAS, to regulate the type II interferon signaling modules M32 and M29, suggesting a mechanism through which it might mediate its effect on diverse traits and diseases. Here, formal Mendelian randomization analysis was not deemed appropriate due to the known pleiotropy at this locus. Our results are in line with the previously described functional effects of the *SH2B3* rs3184504 variant on proinflammatory cytokine production [62]. The *SH2B3* rs3184504 variant has also been associated with the expression of multiple genes [63], and *SH2B3* in *cis* and 14 other genes in *trans*, including *GBP2* and *UBE2L6* from module M32 and *GBP4* and *STAT1* from module M29. Thus, this locus seems to be a transcriptional master regulator of immune pathways, while at the same time exhibiting pleiotropic effects on a number of traits and diseases, including T2D. Many of the module-QTL loci overlap with GWAS hits for immune-related phenotypes, suggesting that the modules described here might be of importance in the context of inflammatory diseases. Similar analyses should be performed for co-expression modules in other more T2D-relevant tissues to provide further insight into the causal networks underlying T2D aetiology. Similarly, network rewiring in T2D might be more strongly detectable in other tissues than blood, although we did observe changes in module connectivity with diabetes status for Rho signaling and erythrocyte pathway modules.

To conclude, we have performed a large-scale analysis of whole blood transcriptomics in the context of metabolic traits and T2D. By exploring associations with clinical traits, trans-omics associations, genetic enrichment and module rewiring by disease state, we provide a comprehensive view of the relationship between whole blood co-expression modules and metabolic health in non-diabetic individuals and T2D patients and highlight novel candidates for further studies.

## Supplementary information

**Additional file 1.** Supplementary Figures. This file contains Fig. S1 – S13.

**Additional file 2.** Supplementary Methods. This file contains methods descriptions for omics data generation and preprocessing.

**Additional file 3.** Supplementary Tables. This file contains Tables. S1 – S14.

## Data Availability

Due to the type of consent provided by study participants and the ethical approvals for this study, individual-level clinical and omics data from IMI-DIRECT cohorts cannot be transferred from the centralized IMI-DIRECT repository. Requests for access to IMI-DIRECT data, including data presented here, can be made to DIRECTdataaccess@Dundee.ac.uk. Requestors will be provided with information and assistance on how data can be accessed via the DIRECT Computerome secure analysis platform following submission of appropriate documentation. The IMI-DIRECT data access policy is available from www.direct-diabetes.org.
