# Supplementary material for: Whole blood co-expression modules associate with metabolic traits and type 2 diabetes: an IMI-DIRECT study
Source: Genome Med. 2020 Dec 1;12:109. doi: 10.1186/s13073-020-00806-6 (PMC7708171; doi:10.1186/s13073-020-00806-6)
Supplement: Supplementary file 1 — Additional file 1. Supplementary Figures. This file contains Fig. S1 – S13. [file 13073_2020_806_MOESM1_ESM.pdf]

Supplementary Figures

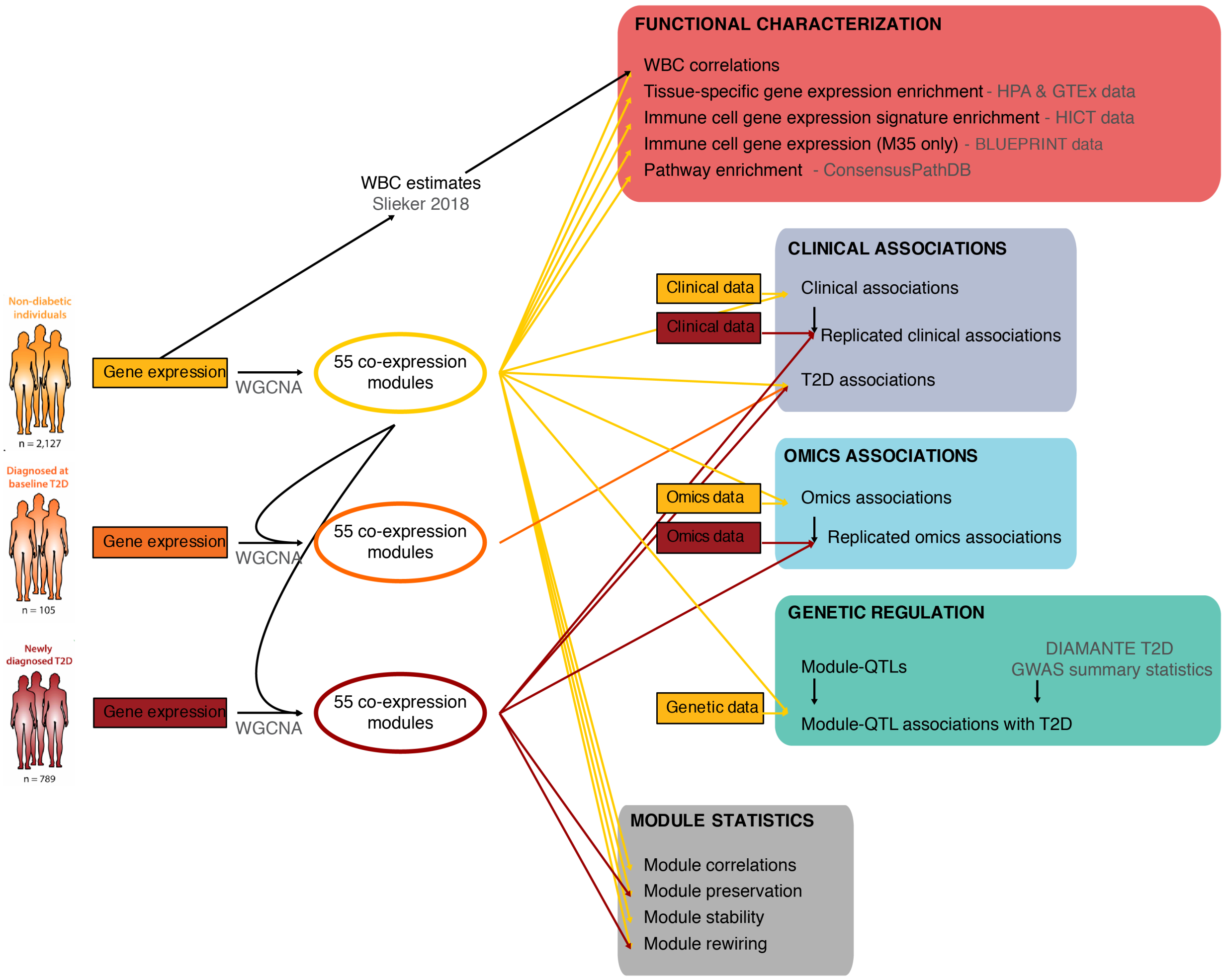

Fig. S1 – An overview of the workflow applied in the current study. Whole blood gene expression data from each of the three included study cohorts were used to define co-expression modules that were further characterized in terms of function and tissue/cell-type expression, clinical associations, omics associations, genetic regulation and links to T2D genetics, and various module statistics related to stability, preservation and rewiring. WBC, white blood cell; WGCNA, weighted gene co-expression network analysis.

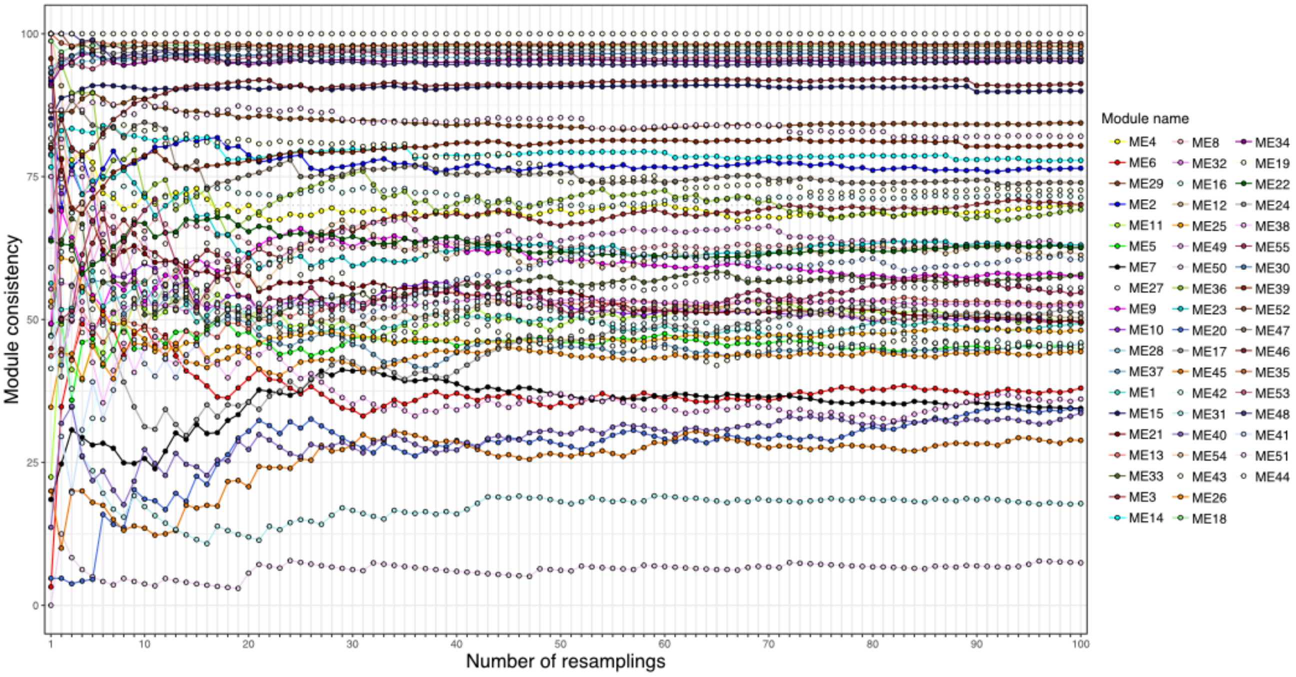

Fig. S2 – Module stability for the 55 modules as a function of number of resamplings, calculated using the non-diabetic cohort (n = 2,127). As can be seen from the figure, the value for most modules stabilizes at around ~40 resamplings, indicating that 100 resamplings are more than sufficient to provide an accurate estimate of module stability.

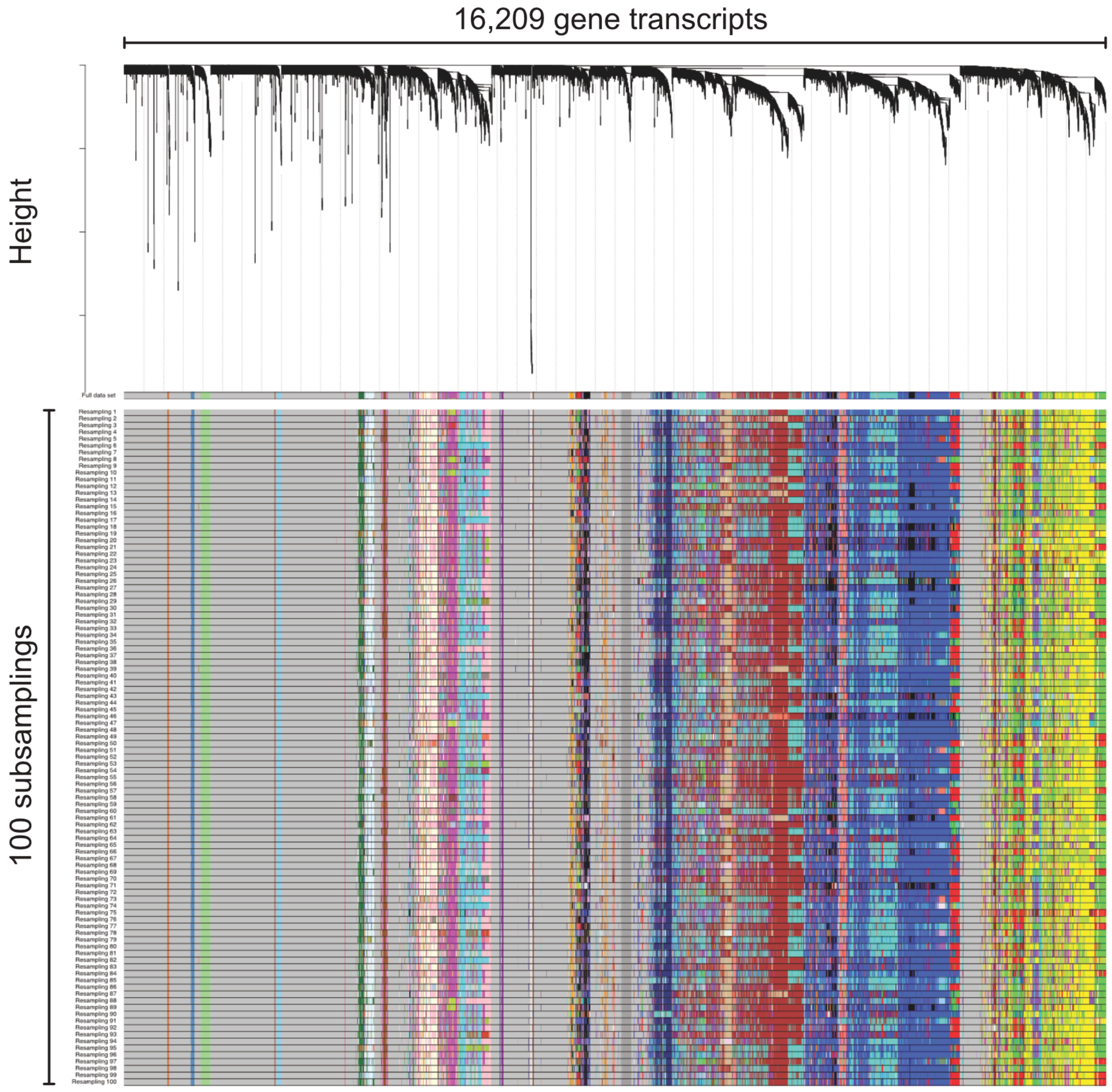

Fig. S3 – The transcriptomic data in the non-diabetic cohort (n = 2,127) was clustered into 55 modules as indicated by the dendrogram and the colored bar below (top panel), in addition to genes that fell outside of any given module (grey) (see Methods for details). The bottom panel illustrates the results from 100 runs of subsampling used to calculate module stability.

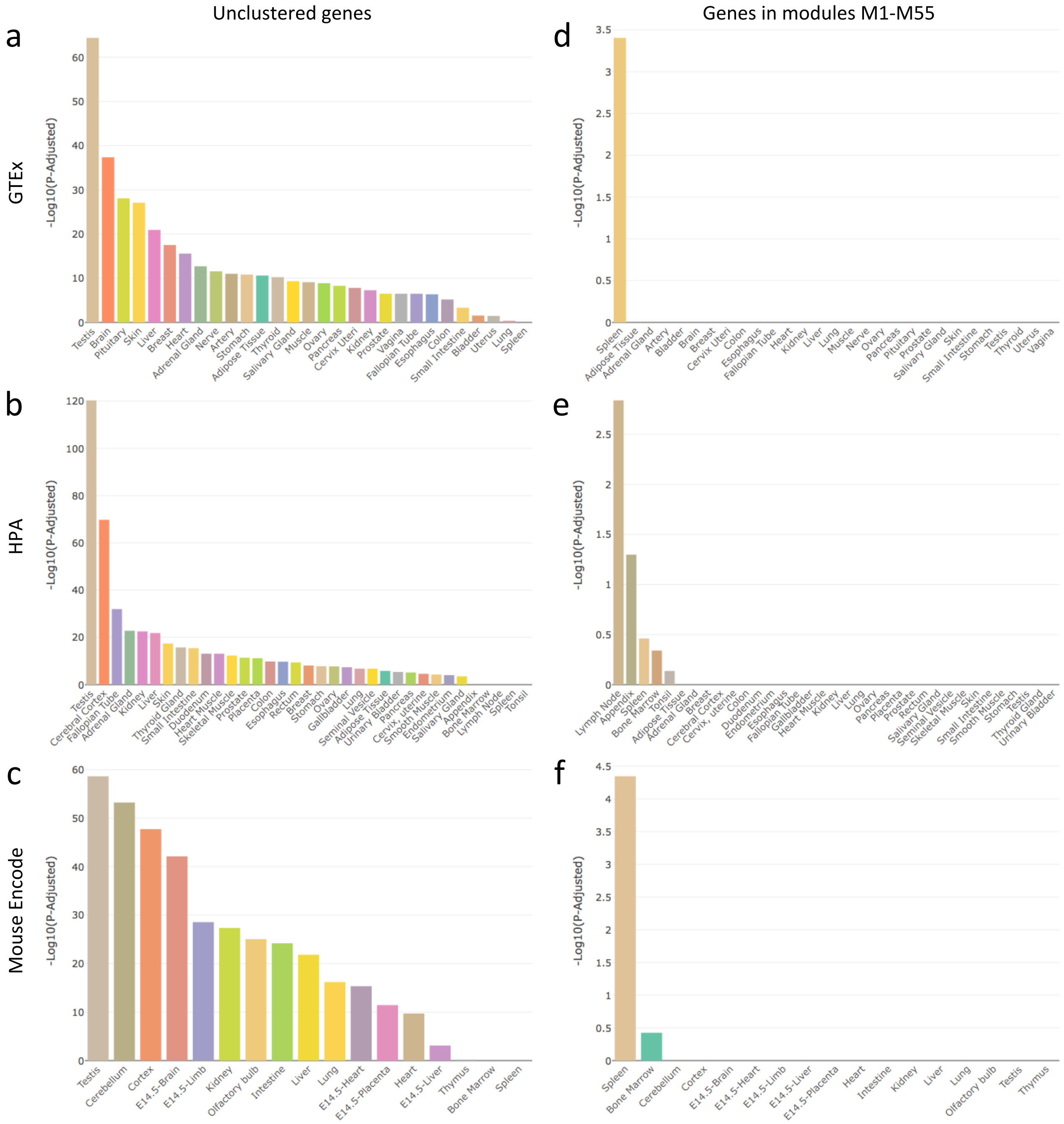

Fig. S4 – Enrichment of tissue-specific genes for **a-c)** unclustered genes (M0) and **d-f)** genes clustered into modules M1-M55, compared to the full set of 16,209 genes included in the transcriptomics analysis. The enrichment analysis was performed using TissueEnrich and based on transcriptomics data across tissues from a),d) GTEx, b),e) the Human Protein Atlas and c),f) Mouse Encode.

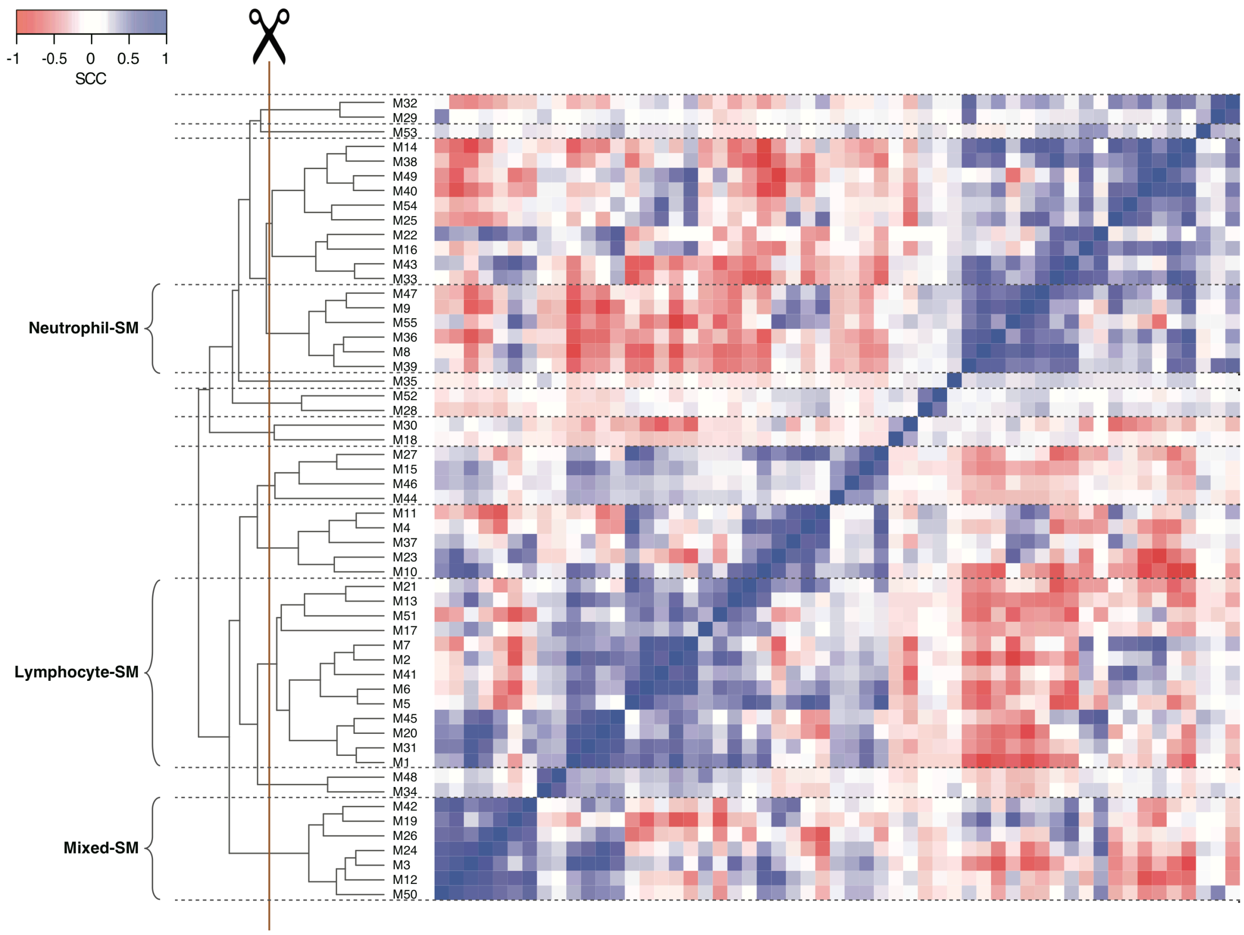

Fig. S5 – Internal correlations between the transcriptomic modules reveal a number of related super-modules. The brown vertical line indicates where the dendrogram is cut. The applied clustering approach was tailored to only group positively correlated genes. Consequently, genes with negative correlation will end up in two different and negatively correlated clusters, causing the inverse relationships observed between many pairs of clusters. SCC: Spearman's correlation coefficient.

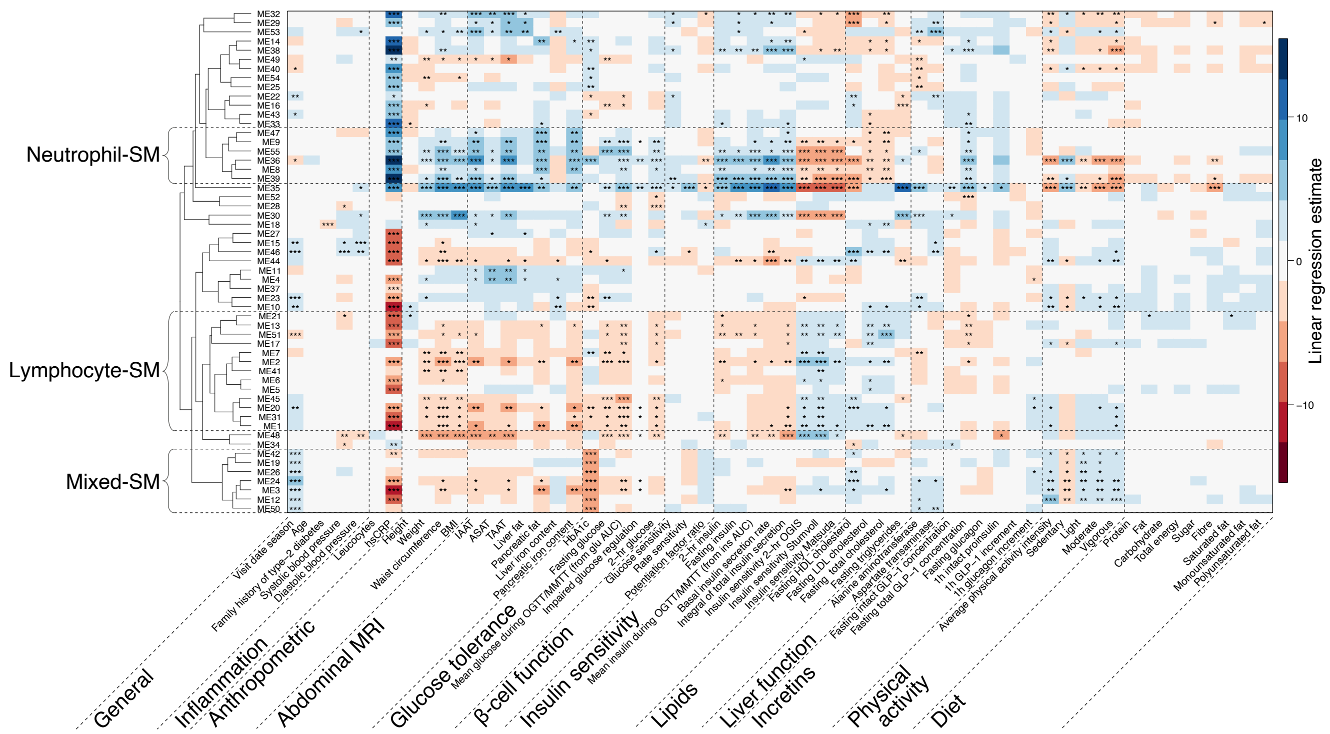

Fig. S6 – Associations between the 55 transcriptomic modules and the full set of baseline clinical traits tested. The heatmap colors denote the linear regression estimates, where all phenotypes have previously been rank normal transformed and residualised for age, sex, center and technical covariates. Stars indicate statistical significance as such: ***FDR < 0.001, **FDR < 0.01, *FDR < 0.05.

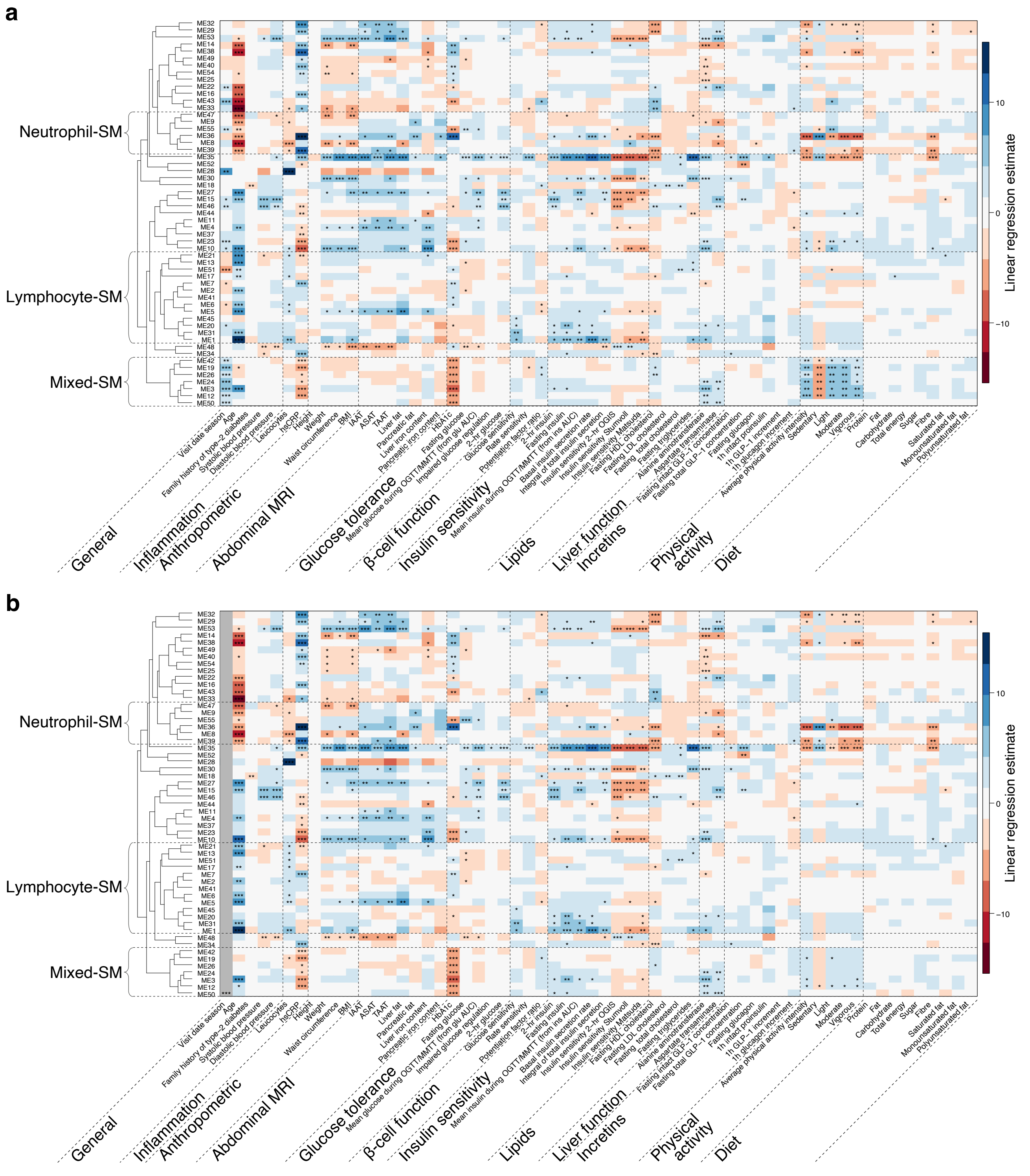

Fig. S7 – Module association with baseline clinical traits, adjusted for **a)** WBC-estimates and **b)** WBC-estimates and season, in addition to age, sex and study center. Many associations are attenuated compared to **Additional file 1: Fig. S6**. The heatmap colors denote the linear regression estimates, where all phenotypes have previously been rank normal transformed. Stars indicate statistical significance as such: ***FDR < 0.001, **FDR < 0.01, *FDR < 0.05.

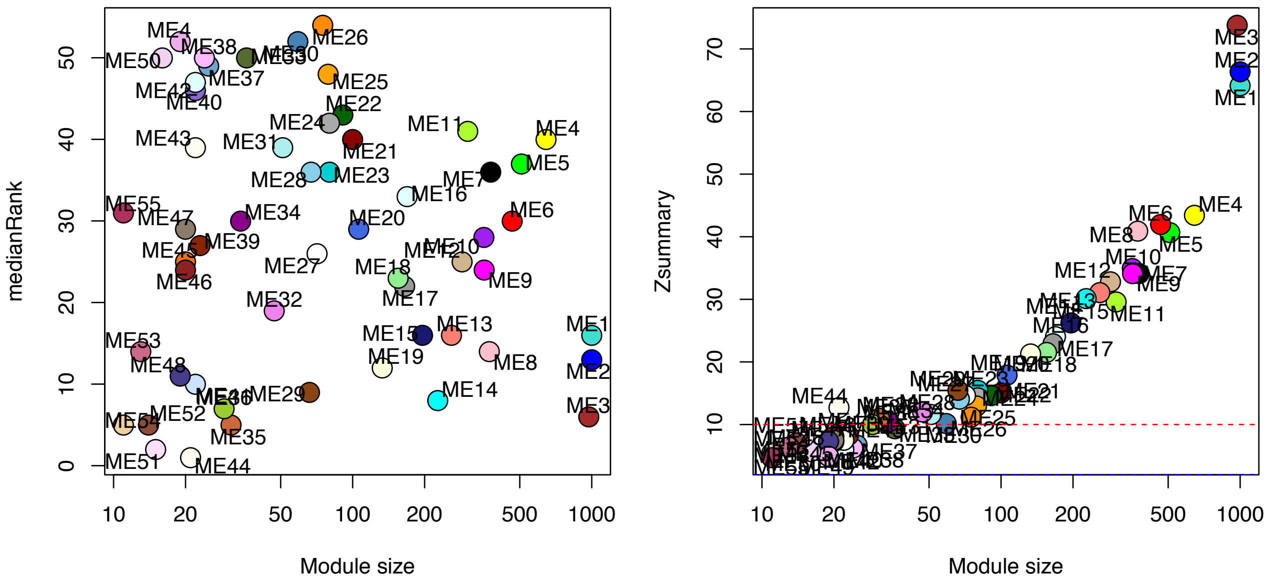

Fig. S8 – Module preservation between the non-diabetic (n = 2,127) and ND-T2D (n = 789) cohorts. Preservation of the transcriptomic modules (as defined in the non-diabetic cohort) are tested in the ND-T2D cohort (see **Methods** for details) and the resulting medianRank (**a**) and Zsummary (**b**) for each module are plotted against module size. The medianRank depicts the relative preservation of modules with lower median ranks indicating stronger tendencies for preservation, while the summary statistic Zsummary describe the absolute preservation, with Zsummary > 10, strong evidence for preservation; 2 < Zsummary < 10, weak to moderate evidence for preservation; Zsummary < 2, no evidence for preservation [37].

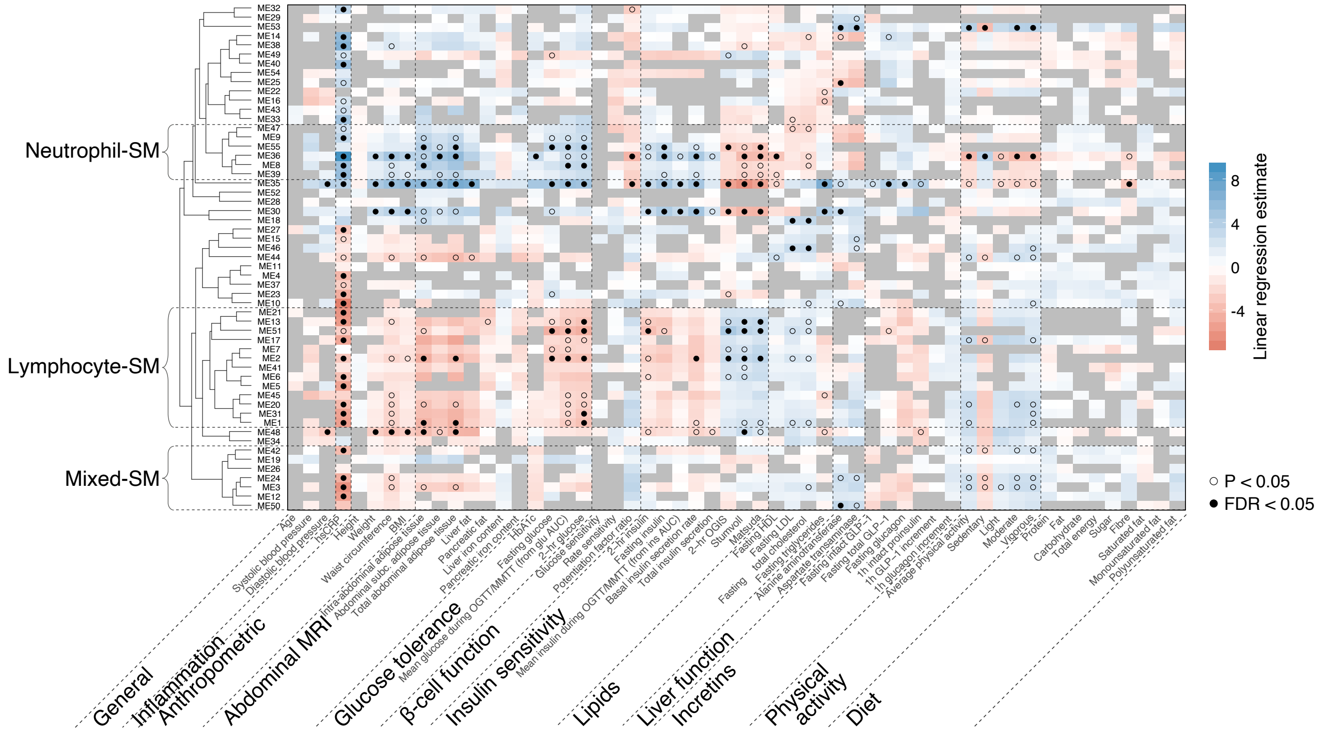

Fig. S9 – Transcriptomic module (as defined in the non-diabetic cohort) associations with baseline clinical traits in 789 newly diagnosed T2D patients (ND-T2D). Directionally consistent associations compared to those observed in the non-diabetic cohort (**Additional file 1: Fig. S6**) are colored while inconsistent associations are shown in grey. The overlayed shapes denote directionally consistent associations that were significant (FDR < 0.05) in the non-diabetic cohort and where unfilled circle: nominally significant (P < 0.05) in the ND-T2D cohort, filled circle: significant (FDR < 0.05) in the ND-T2D cohort.

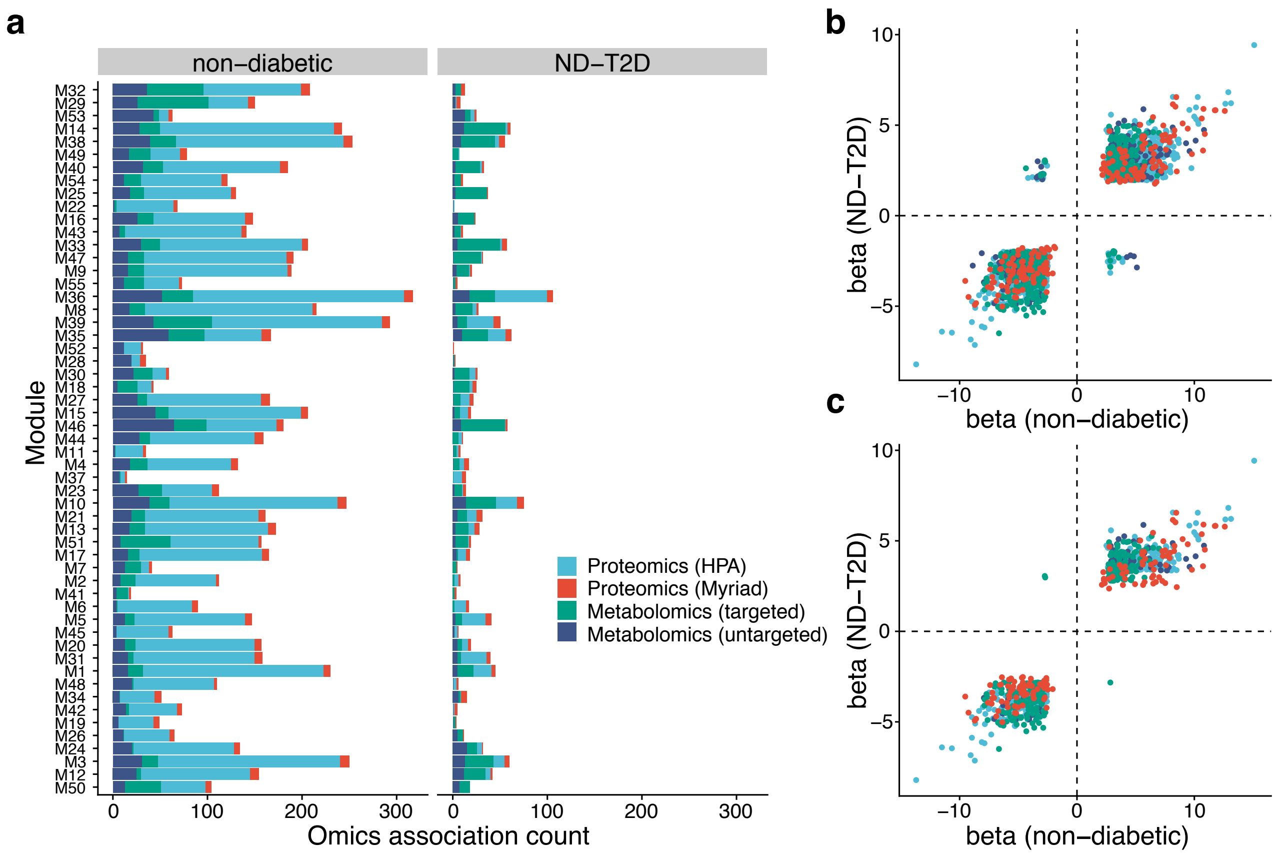

Fig. S10 – **a)** Number of significant (FDR < 0.05) transcriptomic module associations with proteomics and metabolomics data observed in the non-diabetic (n = 2,127) and the ND-T2D (n = 789) cohorts from linear regression analysis. **b-c)** Comparison of omics association beta values between the non-diabetic and ND-T2D cohorts for **b)** all associations that were significant (FDR < 0.05) in the non-diabetic cohort and nominally significant (P < 0.05) in the ND-T2D cohort and **c)** those that were significant (FDR < 0.05) in both cohorts. ND-T2D, newly diagnosed T2D.

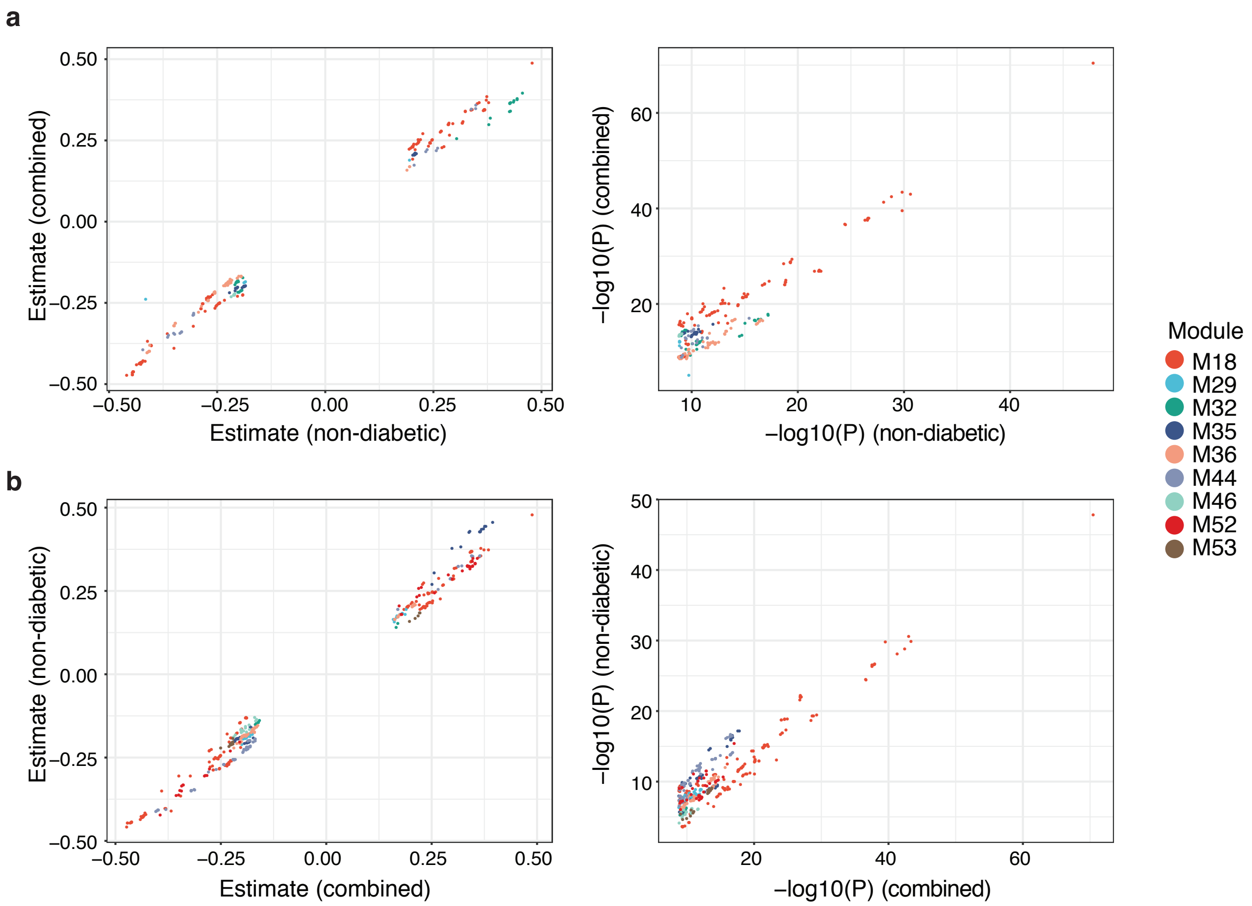

Fig. S11 – Comparison of observed module-QTL effect size and P-values in the non-diabetic cohort (n = 2,127) versus the combined sample of non-diabetic and ND-T2D cohorts (n = 2,914), for significant module-QTLs identified in **a)** the non-diabetic cohort only (n = 2,127) or **b)** the combined sample of non-diabetic and ND-T2D cohorts (n = 2,914).

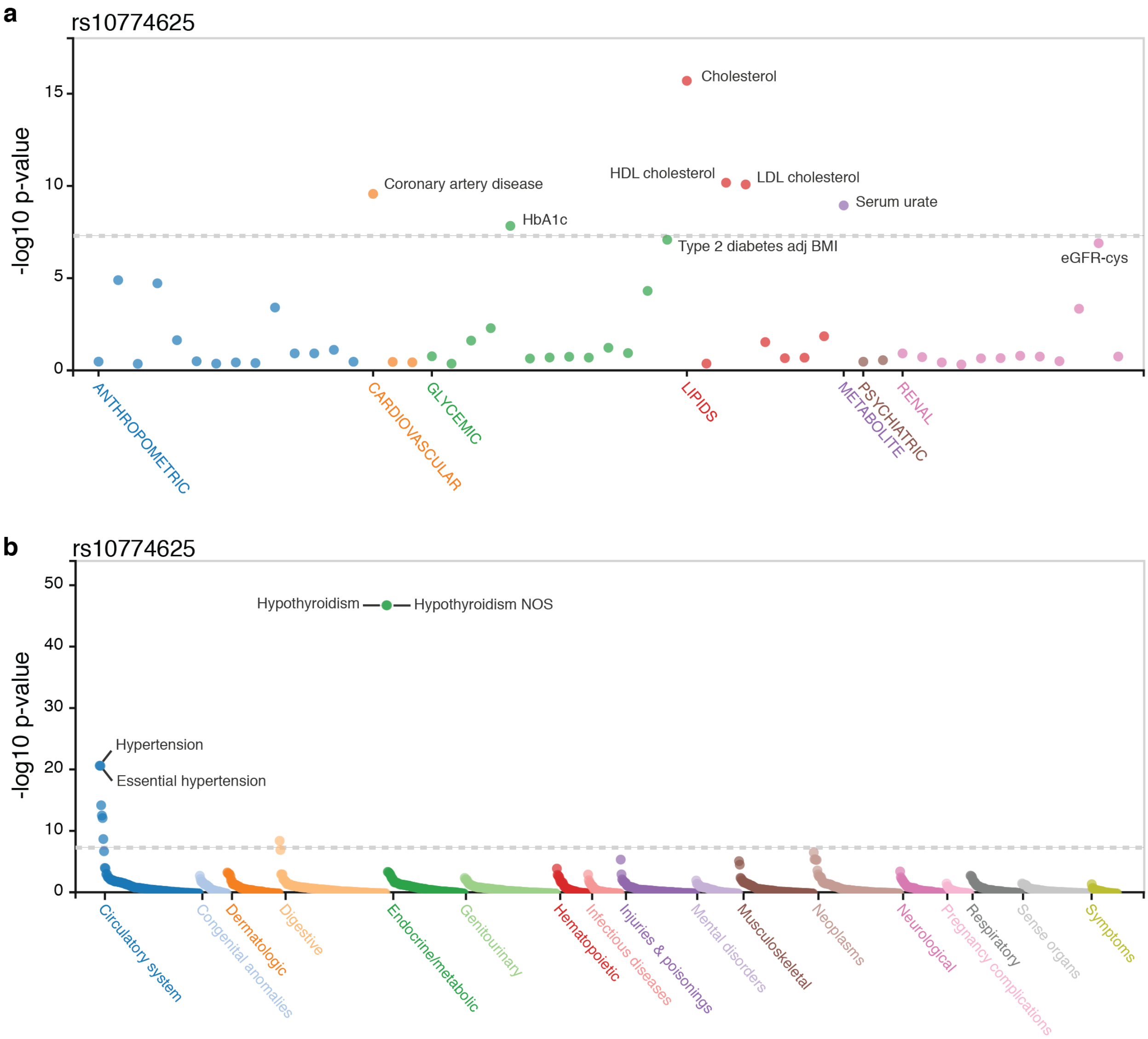

Fig. S12 – Phewas plots for the lead M32 variant rs10774625 adapted from the T2D Knowledge Portal (http://www.type2diabetesgenetics.org) showing GWAS associations from **a)** selected consortia and **b)** UK Biobank. NOS, not otherwise specified.

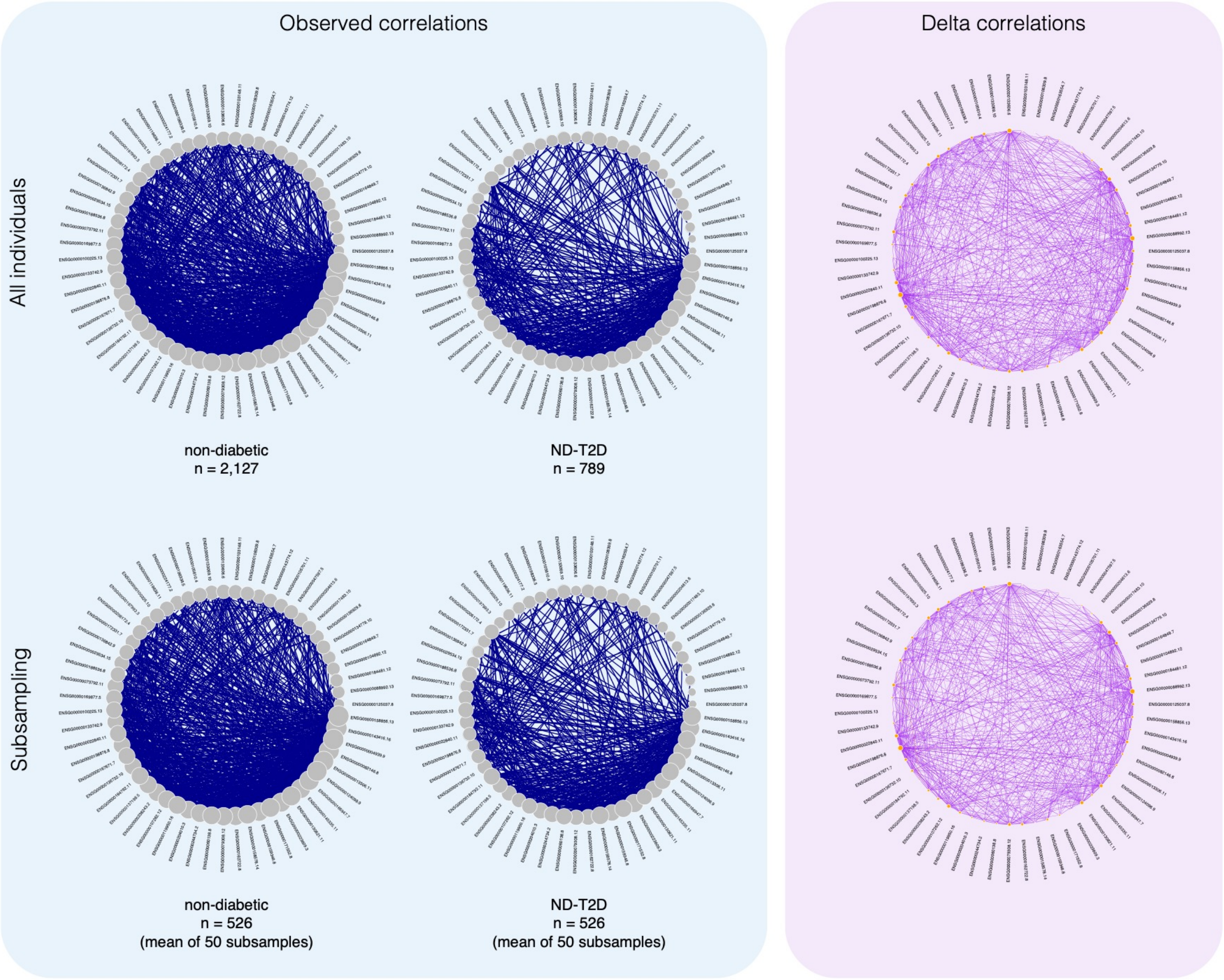

Fig. S13 – Module rewiring for module M30. The blue panel illustrates the correlation patterns between all pairs of the 23 genes constituting module M30, calculated using the non-diabetic (left) or ND-T2D (right) cohort, respectively, and showing results for all individuals in both cohorts (top) and the average of 50 random samples of same size in both cohorts (bottom). Only edges with an absolute biweight midcorrelation coefficient > 0.5 are shown, where blue indicates positive and red negative correlation coefficients. The purple panel shows the difference in correlation coefficients between the non-diabetic and ND-T2D cohorts, where only edges with an absolute difference of correlation coefficients > 0.1 are shown. Nodes are shown in the same order in all six plots and ordered by decreasing 'centrality' in the full non-diabetic cohort (top left) network, defined as average absolute Spearman correlation with all other genes.
