# Supplementary material for: Whole blood co-expression modules associate with metabolic traits and type 2 diabetes: an IMI-DIRECT study
Source: Genome Med. 2020 Dec 1;12:109. doi: 10.1186/s13073-020-00806-6 (PMC7708171; doi:10.1186/s13073-020-00806-6)
Supplement: Supplementary file 2 — Additional file 2. Supplementary Methods. This file contains methods descriptions for omics data generation and preprocessing. [file 13073_2020_806_MOESM2_ESM.pdf]

Supplementary Methods

Transcriptomics 1
Genetics 3
Proteomics 4
Metabolomics 6
Supplementary references 9

Transcriptomics

Sequence generation

For the study of transcriptomic profiles of participants at high risk of T2D and newly diagnosed T2D patients, 3,095 samples of mRNA from whole blood samples were processed for RNA-sequencing. Concentration of mRNA per samples was assessed using the Qubit2.0 from Invitrogen. The quality of the samples was then assessed using the TapeStation Software (A.01.04) with an RNA Screen Tape from Agilent to check the mRNA quality on gel. 29 samples were discarded at this point due to low mRNA quality. The remaining samples were processed and sequencing libraries were prepared. Quality of the libraries was evaluated using Qubit and TapeStation using DNA1000 Screen Tape. One sample was discarded after library preparation due to low quality. The remaining samples (3,065) were placed in Flow cell PE using the cBOT system from Illumina. The samples were then sequenced on the Illumina HiSec2000 platform using 49 bp paired-end reads.

Read mapping and exon quantifications

The 49-bp sequenced paired-end reads were mapped to the GRCh37 reference genome (Lander et al., 2001) with GEMTools 1.7.1 (Marco-Sola et al., 2012). Exon quantifications were calculated for all elements annotated on GENCODE v19 (Harrow et al., 2012). All overlapping exons of a gene were merged into meta-exons with identifier of type ENSG000001.1_exon.start.pos_exon.end.pos. Read counts over these elements used paired-end reads if their both ends have a quality score >= 150, a total mismatch <= 5 (5 mismatches max in 2x49pb) and if they are in proper orientation. We filtered transcripts from genes that were not protein coding, linRNA or processed transcripts if they overlap in the opposite strand with protein coding gene sand lincRNA genes. For split reads, we counted the exon overlap of each split fragment, and added counts per read as 1/(number of overlapping bases per exon). For genes quantification, FPKM values were calculated.

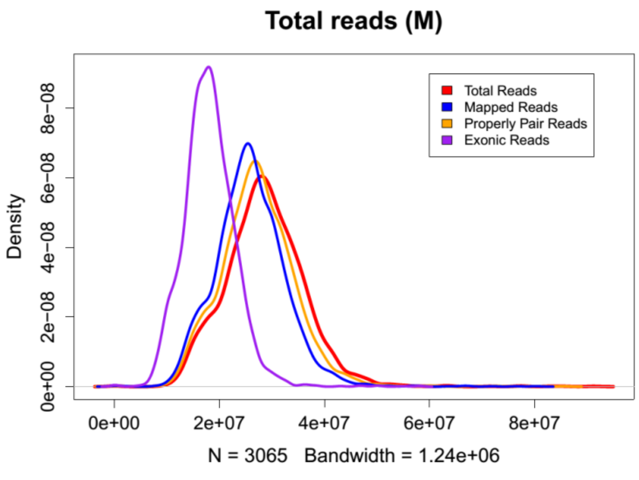

Figure 1

Sample quality assessment and filtering
Samples with a total number of exonic reads lower than 5e+06 reads or with a proportion of exonic reads over the total number of reads lower than 20% were considered of low quality (4 samples excluded, Figure 1).

Identification of sample mix-ups and labelling errors is possible when genotypes are available ('t Hoen et al., 2013). A total of 3,057 samples had genotypes available. For each samples, we tested the heterozygous sites in DNA genotypes for expression of both alleles in the RNAseq data. Mixed-up or mislabelled samples show lower levels of expression of both alleles. Using the function *match* from the suite QTLtools (Fort et al., 2017), we tested each expression profile (BAM files) against all imputed genotypes from DIRECT (Project Release v2, February 2017) to identify the best matching expression-genotype pair.

The analysis identified 201 samples with mismatch between expression and genotypes. Of those samples 137 could be corrected as we identified another genotype with a good match within the data set. Other 4 samples had low quality or were mixes of RNAseq samples and would not be identified with confidence. For 60 samples we would not find a suitable match between expression and the available genotypes. After correcting samples swaps, 3 individuals were found to have duplicated RNAseq data. To confirm the correct assignment of the matched DNA/RNA samples and recovered failed genotypes during QC we re-genotyped samples from 96 individuals. After repeating the genotypes calling and quality assessment, we confirmed the correct alignment for those samples. We also recovered samples for which genotypes were not available in the first round, making a total 162 recovered samples. The total number of European samples with RNAseq-genotype pairing data after QC was 3,029.

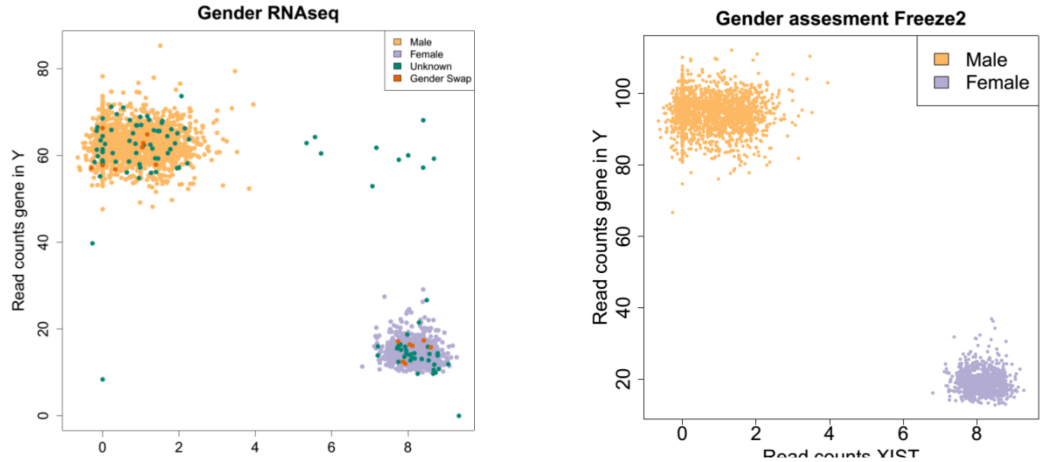

Figure 2

Gender identification in RNAseq compare expression levels of genes in the autosomal region for chromosome Y and the expression of the *XIST* gene in chromosome X (Figure 2). To confirm gender information and validate the identity of the sequence data, we compared the gender provided by clinical reports and the gender identify by genotype data with the gender identify from RNAseq data. The analysis identified 887 females and 2,142 males for both pre- and diabetic patients. For 13 samples the gender analysis in expression was inconclusive, those were due to small mixes of RNAseq samples, low RNAseq quality or unreported biological factors.

Filtering

Genes and exons with more than 50% of zero reads were removed from the study. To ensure enough individuals with no zero reads in both the non- and the diabetic study we filtered the exons and genes with zero reads in more than 50% non-diabetic individuals and in more than 50% diabetic individuals as defined by the cohort at screening. Finally, exons and genes from chromosome Y, mitochondria, and level 3 annotation, as encoded by Gencode v19, were removed from further analysis.

Software

At the time of the study, custom scripts were used to for any intermediate step and quantification of exon and genes, as well as quality assessment of the samples. The same pipeline can now be found in (Delaneau et al., 2017) as part of QTLtools.

Genetics

DNA extraction, genotyping, and quality control

DNA extraction of participants at high risk of T2D and newly diagnosed T2D patients was carried out using Maxwell 16 Blood DNA purification kits and a Maxwell 16 semi-automated nucleic acid purification system (Promega). Genotyping was conducted in two tranches using the Illumina HumanCore array (HCE24 v1.0) and genotypes were called using Illumina's GenCall algorithm.

In the first round, 3102 samples were genotyped and samples were excluded for any of the following reasons: call rate <97%; low or excess mean heterozygosity; gender discordance; duplicates; and monozygosity. Genotyping quality control was then performed to provide high-quality genotype data for downstream analyses using the following criteria: call rate <99%; deviation from Hardy-Weinberg equilibrium (exact $p<0.001$); variants not mapped to human genome build GRCh37; and variants with duplicate chromosome positions. We performed an additional quality control step to identify plausible sample swaps and/or sample labelling errors utilizing available RNAseq data on genotyped samples. Both genotype and RNAseq data was available for 3,057 samples. For each of these samples, we identified the best matching expression-genotype pair using expression profile and genotypes (see transcriptomics section above). We identified 201 instances of mismatch between genotype and expression sample identifiers. For these samples, we examined the reported and (genotype) derived gender and traced back the samples through each step involved in their acquisition, extraction, and genotyping. After accounting for manipulation errors through these steps and re-mapping samples to correct identifiers, we recovered 137 of the 201 samples.

We carried out a second round of genotyping of 96 samples to: (i) confirm the correct assignment of the matched DNA/RNA samples; and (ii) recover genotyped samples that failed quality control due to low genotype rate. We repeated quality assessment of these samples as described above and then combined samples from both genotype tranches and conducted another round of sample and variant quality control using the same criteria as above. We also confirmed the correct alignment for all DNA/RNA samples fixed above. We used autosomal variants with MAF>1% that passed quality control to construct axes of genetic variation using principal components analysis implemented in PLINK software to

identify ethnic outliers defined as non-European ancestry using the 1000 Genome Project samples as reference (Auton et al., 2015). A total of 3,029 European samples with genotype-RNAseq pairing passed the final quality control.

Pre-phasing and imputation

All samples passing quality control were taken forward for pre-phasing and imputation. Before pre-phasing variants were removed if: (i) allele frequencies differed from those for European ancestry haplotypes from the 1000 Genome reference panel by more than 20%; (ii) AT/GC variants had MAF>40% because of potential undetected errors in strand alignment; or (iii) MAF<1% because of difficulties in calling rare variants. After these exclusions, a total of 273,568 variants remained. Samples were first pre-phased using SHAPEIT1 (version v2.r790) and then imputed up to the 1000 Genome Project reference panel (phase 3, October 2014 release; X chromosome, phase 3, August 2015 release) using IMPUTEv2.3 (Delaneau et al., 2013; Howie et al., 2009).

Proteomics

Antibody and target selection

A Biomarker Task Force was formed with the DIRECT consortium to select proteins of interest for plasma analysis. This led to a list of 442 protein candidates with potential links to T2D from literature mining, protein and gene expression in beta cells and islets, proteins of clinical relevance, GWAS and eQTL studies, previous use of antibodies in the applied assay, as well as a protein-protein interaction network analysis. Antibodies were chosen based on availability from the Human Protein Atlas (HPA) (Nilsson et al., 2005; Uhlén et al., 2005, 2015). We found 779 HPA antibodies for 385 out of 442 proteins. Prioritizing proteins for which more than one antibody was accessible, 640 antibodies for 252 proteins were selected for antibody performance tests. The antibodies were applied to an assay with a subset of 340 plasma samples (256 from the non-diabetic cohort and 84 from the T2D cohort) using the assay procedure as described below to test the property of the antibodies in the contexts of these samples. Antibodies were excluded from further studies if 1) signal intensities were obtained lower than the internal negative control (6 HPAs were excluded) and 2) the variance in signal intensities across samples were smaller than the control antibody (127 HPAs were excluded). A set of 380 antibodies targeting 265 proteins was selected for subsequent analyses.

Generation of antibody bead arrays

All selected HPA antibodies were coupled to beads to generate antibody bead arrays in suspension (as described below). As assay controls, antibodies against albumin (DAKO) and anti-human IgG (Jackson ImmunoResearch) were included, as well as beads coupled with normal rabbit IgG to resemble the scaffold of HPA antibodies. One bead identity did not include any protein during the coupling procedure (denoted bare beads).

Antibodies were coupled to carboxylated magnetic beads (MagPlex-C, Luminex Corp.) in accordance to previously developed protocols (Drobin et al., 2013; Schwenk et al., 2008, 2010). Briefly, 5×10^5 beads per bead identity were distributed in 96-well microtiter plates (Greiner BioOne). Beads were initially washed and re-suspended in phosphate buffer (0.1 M

NaH2PO4, pH 6.2) using a plate washer (EL406, Biotek). Bead activation was performed by adding 0.5 mg 1-ethyl-3(3-dimethylamino-propyl) carbodiimide (Pierce) and 0.5 mg N-hydroxysuccinimide (Pierce) dissolved in 100 µl phosphate buffer. After 20 min incubation at 650 rpm on a plate shaker (Grant Bio), beads were washed with 0.1 M 2-(N-morpholino)ethanesulfonic acid (MES) buffer (pH 4.5) on a plate washer (EL406, Biotek). 1.6 µg of each antibody had been pre-diluted in 100 µl of MES buffer by a liquid handling system (EVO150, Tecan) and were subsequently added to the activated beads. After 2 h incubation at RT, beads were washed 3× in PBS-T (1 × PBS, 0.05% Tween20). Next, 50 µl of a protein blocking buffer (Blocking Reagent for ELISA, Roche Applied Science) supplemented with 0.1% (v/v) ProClin (Sigma-Aldrich) was added for an overnight incubation at 4°C. Finally, mixing the 384 different bead identities resulted in 384-plexed suspension bead arrays that were stored at 4°C in the dark until further use. R-Phycoerythrin-conjugated donkey anti-rabbit IgG antibody (Jackson ImmunoResearch) was utilized to confirm an efficient coupling of antibodies.

Experimental design
Samples from the four different centers were distributed across microtiter plates via a supervised randomization procedure. The plate layouts were carefully designed to minimize and equalize the time that each sample was placed at room temperature during the transferring of samples into plates. To achieve this, plasma samples were in designated orders, thawed over night at 4°C, centrifuged for 10 min at 3,000 × g, and distributed into the designed plate layout by the use of a liquid handling system (Freedom EVO150, TECAN). After sample randomization, the randomized 96-well microtiter plates were stored at -80°C until further use.

Antibody beads array assays
Plasma samples in randomized plate layouts were thawed at 4°C and centrifuged for 10 min at 3,000 × g. Three microliters of each sample were diluted in 22 µl of 1x PBS using a liquid handler (SELMA, CyBio). Biotinylation of diluted plasma was performed as previously described (Schwenk et al., 2008). Briefly, labeling was enabled by a 2 h incubation of samples with a 10-fold molar excess of NHS-PEG4-Biotin (Pierce) at 4°C. The biotinylation reaction was quenched by the addition of 0.5 M Tris-HCl (pH 8.0) with a 250-fold molar excess over biotin. After 20 min incubation with Tris-HCl at 4°C, samples were stored at -80°C until usage. Biotinylated samples were diluted 1:50 using a liquid handler (SELMA, CyBio) in assay buffer.

The assay buffer was composed of 0.5% (w/v) polyvinylalcohol and 0.8% (w/v) polyvinylpyrrolidone (Sigma) in 0.1% (w/v) casein (Sigma-Aldrich) in PBS (PVXC) supplemented with 0.5 mg/ml rabbit IgG (Bethyl). Prior incubation with beads, samples were heat-treated at 56°C for 30 min in a water bath (TW8, Julabo) followed by 15 min cooling at RT. 5 µl of the antibody suspension bead array (~200 beads per bead identity) was distributed into 384-well microtiter plates (Greiner BioOne). 45 µl of heat-treated samples were then added to each bead plate by the use of a liquid handler (SELMA, CyBio). After an overnight incubation at RT on a shaking table (Grant) beads were washed with 3 × 50 µl PBS-T on a plate washer (EL406, Biotek). Samples were cross-linked with 0.4% paraformaldehyde in PBS-T for 10 min, washed 3 × 50 µl PBS-T and 50 µl of 0.5 lg/ml R-phycoerythrin labeled

streptavidin (Invitrogen) in PBS-T was added. After 20 min incubation, beads were finally washed 3 × 50 µl PBS-T and resuspended in 50 µl PBS-T for measurement in a FlexMap3D instrument (Luminex Corp.). At least 50 bead counts were counted per bead identity. The median fluorescence intensity (MFI) was used to represent the relative amount of target protein binding to each of the antibody-coupled bead identity.

Data quality assessment
The obtained data was evaluated based on intensity levels and three antibodies were excluded from further analysis as the median MFI were below negative control antibodies (bare and rabbit IgG beads). Because one stock solution of mixed beads was created and aliquoted into each assay plate, other experimental errors were linked to the procedure for individual samples. Thus, eight samples were flagged that seemingly failed. Such samples were those 1) that had median values of MFIs ± 2 SD or below the median of control measurement without any sample (buffer only), and 2) that were identified as outliers using Robust PCA using the 'rrcov' R package (version 1.4-3) (Hubert et al., 2005). The cutoff probability values in an outlier diagnostic plot were set to 0.001 for both score and orthogonal distances. The samples deviating beyond the cutoffs in both distance coordinates were classified as outliers, setting alpha, the proportional tolerance, to 0.9. The remaining data set was denoted as annotated.

Data pre-processing
The annotated data was processed by probabilistic quotient normalization (Dieterle et al., 2006) for sample-by-sample variation within the samples collected in same center and assay plates analyzed on the same day. The variation introduced by multiple assay plates was minimized by Multi-MA normalization (Hong et al., 2016). Inverse normal transformation was then applied to the normalized data to reduce the effects of outliers.

Other protein measurements
Additional proteins were analyzed in randomized samples using the services from Myriad RBM (Myriad GmbH, Germany) and for hsCRP (MLM Medical Labs GmbH, Germany).

Metabolomics

Targeted metabolomics – Biocrates Absolute IDQ™ p150 kit
Plasma concentrations of 163 metabolites were determined using a FIA-ESI-MS/MS-based targeted metabolomics approach with the Absolute *IDQ*™ p150 kit (BIOCRATES Life Sciences AG, Innsbruck, Austria). The assay allows simultaneous quantification of 163 metabolites out of 10 µL plasma, and includes free carnitine, 40 acylcarnitines (Cx:y), 15 amino acids (Leu and Ile are measured togetheras xLeu), hexoses (sum of hexoses – about 90-95 % glucose), 91 glycerophospholipids (15 lysophosphatidylcholines (lysoPC.Cx:y) and 76 phosphatidylcholines (PC.Cx:y)), and 15 sphingolipids (SM.Cx:yc). The abbreviations Cx:y are used to describe the total number of carbons and double bonds of all chains, respectively. The LODs were set to three times the values of the zero samples (PBS). The LLOQ and ULOQ were determined experimentally by Biocrates. The assay procedures of the Absolute*IDQ*™ p150 kit as well as the metabolite nomenclature have been described in detail previously (Römisch-Margl Helmholtz et al., 2011; Zukunft et al., 2013). Analytical specifications for LOD and evaluated quantification ranges, further LOD for semi-

quantitative measurements, identities of quantitative and semiquantitative metabolites, specificity, potential interferences, linearity, precision and accuracy, reproducibility, and stability were described in Biocrates manual AS-P150.
Sample handling was performed by a Hamilton Microlab STAR™ robot (Hamilton Bonaduz AG, Bonaduz, Switzerland) and a Ultravap nitrogen evaporator (Porvair Sciences, Leatherhead, U.K.), beside standard laboratory equipment. Mass spectrometric analyses were done on an API 4000 triple quadrupole system (Sciex Deutschland GmbH, Darmstadt, Germany) equipped with a 1200 Series HPLC (Agilent Technologies Deutschland GmbH, Böblingen, Germany) and a HTC PAL auto sampler (CTC Analytics, Zwingen, Switzerland) controlled by the software Analyst 1.6.2. Data evaluation for quantification of metabolite concentrations and quality assessment was performed with the software MultiQuant 3.0.1 (Sciex) and the Met*IDQ*™ software package, which is an integral part of the Absolute*IDQ*™ kit. Metabolite concentrations were calculated using internal standards and reported in µM.
In addition to the investigated samples, five aliquots of a pooled reference plasma (Ref_Plasma-Hum_PK3) were analyzed on each kit plate.

Targeted metabolomics – quality control
After data export from Met*IDQ*™, a first technical QC comprising analysis of peak shapes, retention times, and compound identity was performed. In a second QC step, possible batch effects and effects of different phenotypes were investigated using principal component analysis (PCA). Data were corrected for batches. Lower outliers were defined as samples with >33% of metabolite concentrations below 25% quantile – 1.5*IQR. Upper outliers were defined as samples with >33% of metabolite concentrations above 25% quantile + 1.5*IQR. Metabolite traits with too many zero concentration samples and NAs (>50%) were excluded (none). The Coefficient of Variation (CV) was calculated in reference samples for each metabolite over all plates. Metabolite traits with CV>0.25 were excluded. Metabolite traits with > 95% of samples below LOD were marked.

Untargeted metabolomics
Plasma samples were stored at -80°C prior to analysis at Helmholtz Zentrum München, Germany. On the day of extraction, samples were thawed on ice, were randomized, and were distributed into 69 and 25 batches for the non-diabetic and T2D cohort, respectively. A hundred µL of the plasma were pipetted into a 2 mL 96-well plate. In addition to samples from this study, a pooled human reference plasma sample (Seralab, West Sussex, UK) was extracted in the same way as samples of the study and placed on 7 wells of each batch. These samples served as technical replicates throughout the data set to assess process variability. Besides those samples, 100 µL of water was extracted as samples of the study and placed in 6 wells of each 96-well plate to serve as process blanks.

Protein was precipitated and the metabolites in the plasma samples were extracted with 475 µL methanol, containing four recovery standard compounds to monitor the extraction efficiency. After centrifugation, the supernatant was split into 4 aliquots of 100 µL each onto two 96-well microplates. The first 2 aliquots were used for LC-MS/MS analysis in positive and negative electrospray ionization mode. Two further aliquots on the second plate were kept as a reserve. The samples were dried on a TurboVap 96 (Zymark, Sotax, Lörrach, Germany). Prior to LC-MS/MS in positive ion mode, the samples were reconstituted with 50

µL of 0.1% formic acid and those analyzed in negative ion mode with 50 µL of 6.5 mM ammonium bicarbonate, pH 8.0. Reconstitution solvents for both ionization modes contained further internal standards that allowed monitoring of instrument performance and also served as retention reference markers. To minimize human error, liquid handling was performed on a Hamilton Microlab STAR robot (Hamilton Bonaduz AG, Bonaduz, Switzerland).

LC-MS/MS analysis was performed on a linear ion trap LTQ XL mass spectrometer (Thermo Fisher Scientific GmbH, Dreieich, Germany) coupled with a Waters Acquity UPLC system (Waters GmbH, Eschborn, Germany). Two separate columns (2.1 x 100 mm Waters BEH C18 1.7 µm particle) were used for acidic (solvent A: 0.1% formic acid in water, solvent B: 0.1% formic acid in methanol) and for basic (A: 6.5 mM ammonium bicarbonate pH 8.0, B: 6.5 mM ammonium bicarbonate in 95% methanol) mobile phase conditions, optimized for positive and negative electrospray ionization, respectively. After injection of the sample extracts, the columns were developed in a gradient of 99.5% A to 98% B in 11 min run time at 350 µL/min flow rate. The eluent flow was directly connected to the ESI source of the LTQ XL mass spectrometer. Full scan mass spectra (80 – 1000 m/z) and data dependent MS/MS scans with dynamic exclusion were recorded in turns. Metabolites were annotated by curation of the LC-MS/MS data against proprietary Metabolon's chemical database library (Metabolon, Inc., Durham, NC, USA) based on retention index, precursor mass and MS/MS spectra. In this study, 508 (non-diabetic cohort) and 544 (T2D-cohort) metabolites, 330 (non-diabetic cohort) and 341 (T2D-cohort) compounds of known identity (named biochemical) and 178 (non-diabetic cohort) and 203 (T2D-cohort) compounds of unknown structural identity (unnamed biochemical) were identified. The unknown chemicals are indicated by a letter X followed by a number as the compound identifier.

Supplementary references

’t Hoen, P.A.C., Friedländer, M.R., Almlöf, J., Sammeth, M., Pulyakhina, I., Anvar, S.Y., Laros, J.F.J., Buermans, H.P.J., Karlberg, O., Brännvall, M., et al. (2013). Reproducibility of high-throughput mRNA and small RNA sequencing across laboratories. Nat. Biotechnol. *31*, 1015–1022.

Auton, A., Abecasis, G.R., Altshuler, D.M., Durbin, R.M., Abecasis, G.R., Bentley, D.R., Chakravarti, A., Clark, A.G., Donnelly, P., Eichler, E.E., et al. (2015). A global reference for human genetic variation. Nature *526*, 68–74.

Blow, N. (2008). Biochemistry’s new look. Nature *455*, 697–698.

Committee for Medicinal Products for Human Use (CHMP) (2011). Guideline on bioanalytical method validation. EMEA/CHMP/EWP/192217/2009 Rev. 1 Corr. 2.

Delaneau, O., Zagury, J.-F., and Marchini, J. (2013). Improved whole-chromosome phasing for disease and population genetic studies. Nat. Methods *10*, 5–6.

Delaneau, O., Ongen, H., Brown, A.A., Fort, A., Panousis, N.I., and Dermitzakis, E.T. (2017). A complete tool set for molecular QTL discovery and analysis. Nat. Commun. *8*, 15452.

Dieterle, F., Ross, A., Schlotterbeck, G., and Senn, H. (2006). Probabilistic quotient normalization as robust method to account for dilution of complex biological mixtures. Application in 1H NMR metabonomics. Anal. Chem. *78*, 4281–4290.

Do, K.T., Kastenmüller, G., Mook-Kanamori, D.O., Yousri, N.A., Theis, F.J., Suhre, K., and Krumsiek, J. (2015). Network-based approach for analyzing intra- and interfluid metabolite associations in human blood, urine, and saliva. J. Proteome Res. *14*, 1183–1194.

Drobin, K., Nilsson, P., and Schwenk, J.M. (2013). Highly Multiplexed Antibody Suspension Bead Arrays for Plasma Protein Profiling. pp. 137–145.

Fort, A., Panousis, N.I., Garieri, M., Antonarakis, S.E., Lappalainen, T., Dermitzakis, E.T., and Delaneau, O. (2017). MBV: a method to solve sample mislabeling and detect technical bias in large combined genotype and sequencing assay datasets. Bioinformatics *33*, 1895–1897.

Haid, M., Muschet, C., Wahl, S., Römisch-Margl, W., Prehn, C., Möller, G., and Adamski, J. (2018). Long-Term Stability of Human Plasma Metabolites during Storage at −80 °C. J. Proteome Res. *17*, 203–211.

Harrow, J., Frankish, A., Gonzalez, J.M., Tapanari, E., Diekhans, M., Kokocinski, F., Aken, B.L., Barrell, D., Zadissa, A., Searle, S., et al. (2012). GENCODE: The reference human genome annotation for The ENCODE Project. Genome Res. *22*, 1760–1774.

Hong, M.-G., Lee, W., Nilsson, P., Pawitan, Y., and Schwenk, J.M. (2016). Multidimensional Normalization to Minimize Plate Effects of Suspension Bead Array Data. J. Proteome Res. *15*, 3473–3480.

Howie, B.N., Donnelly, P., and Marchini, J. (2009). A flexible and accurate genotype imputation method for the next generation of genome-wide association studies. PLoS Genet. *5*, e1000529.

Hubert, M., Rousseeuw, P.J., and Vanden Branden, K. (2005). ROBPCA: A New Approach to Robust Principal Component Analysis. Technometrics *47*, 64–79.

Lander, E.S., Linton, L.M., Birren, B., Nusbaum, C., Zody, M.C., Baldwin, J., Devon, K., Dewar, K., Doyle, M., FitzHugh, W., et al. (2001). Initial sequencing and analysis of the human genome. Nature *409*, 860–921.

Marco-Sola, S., Sammeth, M., Guigó, R., and Ribeca, P. (2012). The GEM mapper: fast, accurate and versatile alignment by filtration. Nat. Methods *9*, 1185–1188.

Mook-Kanamori, D.O., Selim, M.M.E.-D., Takiddin, A.H., Al-Homsi, H., Al-Mahmoud, K.A.S., Al-Obaidli, A., Zirie, M.A., Rowe, J., Yousri, N.A., Karoly, E.D., et al. (2014). 1,5-Anhydroglucitol in Saliva Is a Noninvasive Marker of Short-Term Glycemic Control. J. Clin. Endocrinol. Metab. *99*, E479–E483.

Nilsson, P., Paavilainen, L., Larsson, K., Odling, J., Sundberg, M., Andersson, A.-C., Kampf, C., Persson, A., Al-Khalili Szigyarto, C., Ottosson, J., et al. (2005). Towards a human proteome atlas: high-throughput generation of mono-specific antibodies for tissue profiling. Proteomics *5*, 4327–4337.

Patti, G.J., Yanes, O., and Siuzdak, G. (2012). Metabolomics: the apogee of the omics trilogy. Nat. Rev. Mol. Cell Biol. *13*, 263–269.

Römisch-Margl Helmholtz, W., München, Z., Suhre, K., Adamski Helmholtz, J., Römisch-Margl, W., Prehn, C., Bogumil, R., Röhring, C., and Adamski, J. (2011). Procedure for tissue sample preparation and metabolite extraction for high-throughput targeted metabolomics Steroid metabolizing enzymes as drug targets View project Genetic and metabolic profiling of elite athletes View project Procedure for tissue sample preparation and metabolite extraction for high-throughput targeted metabolomics.

Schwenk, J.M., Gry, M., Rimini, R., Uhlén, M., and Nilsson, P. (2008). Antibody Suspension Bead Arrays within Serum Proteomics. J. Proteome Res. *7*, 3168–3179.

Schwenk, J.M., Igel, U., Neiman, M., Langen, H., Becker, C., Bjartell, A., Ponten, F., Wiklund, F., Grönberg, H., Nilsson, P., et al. (2010). Toward Next Generation Plasma Profiling via Heat-induced Epitope Retrieval and Array-based Assays. Mol. Cell. Proteomics *9*, 2497–2507.

Uhlén, M., Björling, E., Agaton, C., Szigyarto, C.A.-K., Amini, B., Andersen, E., Andersson, A.-C., Angelidou, P., Asplund, A., Asplund, C., et al. (2005). A human protein atlas for normal and cancer tissues based on antibody proteomics. Mol. Cell. Proteomics *4*, 1920–1932.

Uhlén, M., Fagerberg, L., Hallström, B.M., Lindskog, C., Oksvold, P., Mardinoglu, A., Sivertsson, Å., Kampf, C., Sjöstedt, E., Asplund, A., et al. (2015). Tissue-based map of the human proteome. Science (80-.). *347*, 1260419.

Zukunft, S., Sorgenfrei, M., Prehn, C., Möller, G., and Adamski, J. (2013). Targeted Metabolomics of Dried Blood Spot Extracts. Chromatographia *76*, 1295–1305.
